# Photogeologic Map of the Perseverance Rover Field Site in Jezero Crater Constructed by the Mars 2020 Science Team

**DOI:** 10.1007/s11214-020-00739-x

**Published:** 2020-11-03

**Authors:** Kathryn M. Stack, Nathan R. Williams, Fred Calef, Vivian Z. Sun, Kenneth H. Williford, Kenneth A. Farley, Sigurd Eide, David Flannery, Cory Hughes, Samantha R. Jacob, Linda C. Kah, Forrest Meyen, Antonio Molina, Cathy Quantin Nataf, Melissa Rice, Patrick Russell, Eva Scheller, Christina H. Seeger, William J. Abbey, Jacob B. Adler, Hans Amundsen, Ryan B. Anderson, Stanley M. Angel, Gorka Arana, James Atkins, Megan Barrington, Tor Berger, Rose Borden, Beau Boring, Adrian Brown, Brandi L. Carrier, Pamela Conrad, Henning Dypvik, Sarah A. Fagents, Zachary E. Gallegos, Brad Garczynski, Keenan Golder, Felipe Gomez, Yulia Goreva, Sanjeev Gupta, Svein-Erik Hamran, Taryn Hicks, Eric D. Hinterman, Briony N. Horgan, Joel Hurowitz, Jeffrey R. Johnson, Jeremie Lasue, Rachel E. Kronyak, Yang Liu, Juan Manuel Madariaga, Nicolas Mangold, John McClean, Noah Miklusicak, Daniel Nunes, Corrine Rojas, Kirby Runyon, Nicole Schmitz, Noel Scudder, Emily Shaver, Jason SooHoo, Russell Spaulding, Evan Stanish, Leslie K. Tamppari, Michael M. Tice, Nathalie Turenne, Peter A. Willis, R. Aileen Yingst

**Affiliations:** 1 Jet Propulsion Laboratory, California Institute of Technology, 4800 Oak Grove Drive, Pasadena, CA 91109 USA; 2 California Institute of Technology, Pasadena, CA USA; 3 University of Oslo, Oslo, Norway; 4 Queensland University of Technology, Brisbane, Queensland Australia; 5 Western Washington University, Bellingham, WA USA; 6 Arizona State University, Tempe, AZ USA; 7 University of Tennessee-Knoxville, Knoxville, TN USA; 8 Draper Laboratory, Cambridge, MA USA; 9 Centro de Astrobiología, CAB (INTA, CSIC), Madrid, Spain; 10 University of Lyon, Lyon, France; 11 University of California Los Angeles, Los Angeles, CA USA; 12 Johns Hopkins University, Baltimore, MD USA; 13 Earth and Planetary Exploration Services, Berlin, Germany; 14 USGS-Flagstaff, Flagstaff, AZ USA; 15 University of South Carolina, Columbia, SC USA; 16 University of the Basque Country (UPV/EHU), Leioa, Bizkaia Spain; 17 Cornell University, Ithaca, NY USA; 18 Forsvarets forskingsinstitutt, Kjeller, Norway; 19 Plancius Research, Severna Park, MD USA; 20 Carnegie Institution for Science, Washington, D.C., USA; 21 University of Hawaii at Manoa, Honolulu, HI USA; 22 University of New Mexico, Albuquerque, NM USA; 23 Purdue University, West Lafayette, IN USA; 24 Imperial College of London, London, UK; 25 Massachusetts Institute of Technology, Cambridge, MA USA; 26 State University of New York-Stony Brook, Stony Brook, NY USA; 27 Johns Hopkins Applied Physics Laboratory, Laurel, MD USA; 28 Institut de Recherche en Astrophysique et Planetologie (IRAP), Université de Toulouse, Paul Sabatier, Toulouse, France; 29 Laboratoire Planétologie et Géodynamique, UMR 6112, CNRS, Université de Nantes, Nantes, France; 30 Deutsches Zentrum Fuer Luft- und Raumfahrt E.V., Cologne, Germany; 31 University of Winnipeg, Winnipeg, Manitoba Canada; 32 Texas A&M University, College Station, TX USA; 33 Planetary Science Institute, Tucson, AZ USA

**Keywords:** Mars, Perseverance, Rover, Jezero, Geologic mapping

## Abstract

The Mars 2020 Perseverance rover landing site is located within Jezero crater, a $\sim50~\mbox{km}$ diameter impact crater interpreted to be a Noachian-aged lake basin inside the western edge of the Isidis impact structure. Jezero hosts remnants of a fluvial delta, inlet and outlet valleys, and infill deposits containing diverse carbonate, mafic, and hydrated minerals. Prior to the launch of the Mars 2020 mission, members of the Science Team collaborated to produce a photogeologic map of the Perseverance landing site in Jezero crater. Mapping was performed at a 1:5000 digital map scale using a 25 cm/pixel High Resolution Imaging Science Experiment (HiRISE) orthoimage mosaic base map and a 1 m/pixel HiRISE stereo digital terrain model. Mapped bedrock and surficial units were distinguished by differences in relative brightness, tone, topography, surface texture, and apparent roughness. Mapped bedrock units are generally consistent with those identified in previously published mapping efforts, but this study’s map includes the distribution of surficial deposits and sub-units of the Jezero delta at a higher level of detail than previous studies. This study considers four possible unit correlations to explain the relative age relationships of major units within the map area. Unit correlations include previously published interpretations as well as those that consider more complex interfingering relationships and alternative relative age relationships. The photogeologic map presented here is the foundation for scientific hypothesis development and strategic planning for Perseverance’s exploration of Jezero crater.

**Supplementary Information:**

The online version of this article (10.1007/s11214-020-00739-x) contains supplementary material, which is available to authorized users.

## Introduction

A geologic map is a two-dimensional representation of the three-dimensional geometry of lithostratigraphic units exposed at a planet’s surface. Photogeologic mapping is a proven method of geologic analysis for planets and moons in the Solar System with surfaces that can presently only be studied remotely via robotic spacecraft (Wilhelms 1990). Photogeologic interpretations of flyby and orbiter images of the martian surface have been an important part of Mars science since the Mariner and Viking missions of the 1960s and 1970s (Carr et al. 1983; Scott and Carr 1978; Scott and Tanaka 1986; Greeley and Guest 1987; Tanaka and Scott 1987). Recent high-resolution orbital imaging systems onboard Mars Global Surveyor (MGS), Mars Odyssey, Mars Express, and the Mars Reconnaissance Orbiter (MRO) have revolutionized our understanding of the martian surface, and have led to an updated global geologic map of Mars (Tanaka et al. 2014) and numerous local geologic mapping efforts identifying meter and sub-meter surface detail (e.g., Anderson and Bell 2010; Rice et al. 2013a; Okubo 2014, and Sun and Milliken 2014, among many others).

Photogeologic mapping and interpretations of high-resolution orbiter images have played an important role in landing site selection and in strategic surface exploration planning for recent *in-situ* Mars missions including the Mars Exploration Rovers (MER) Spirit and Opportunity (Arvidson et al. 2006; Golombek et al. 2006; Wray 2009; Wiseman et al. 2010; Crumpler et al. 2011, 2015; Arvidson et al. 2015), the Phoenix Mars Lander (Golombek et al. 2003; Arvidson et al. 2008; Golombek et al. 2008; Seelos et al. 2008), and the Mars Science Laboratory (MSL) Curiosity rover mission (e.g., Milliken et al. 2009; Anderson and Bell 2010; Thomson et al. 2011; Golombek et al. 2012; Grotzinger and Milliken 2012; Rice et al. 2013a). Just prior to Curiosity’s touchdown in Gale crater, the MSL Science Team undertook a group mapping effort of the rover’s landing ellipse using MRO High Resolution Imaging Science Experiment (HiRISE; McEwen et al. 2007) images and digital terrain models (Grotzinger et al. 2014; Calef et al. 2013; Rice et al. 2013b; Sumner et al. 2013). This effort resulted in a detailed photogeologic map of the Curiosity ellipse and surrounding area that was used to guide traverse planning and the selection of the rover’s exploration targets during the MSL prime mission (Grotzinger et al. 2014; Vasavada et al. 2014). This and subsequent mapping efforts in Gale crater, e.g., Fraeman et al. (2016), Stack et al. (2017), have continued to provide geologic context and guidance for planning Curiosity’s traverse and science investigations.

The Mars 2020 Perseverance rover is NASA’s next flagship mission to Mars and the first step in a planned international Mars sample return campaign (Farley et al. this issue). As have previous NASA Mars missions, Mars 2020 benefitted from the engineering and scientific analysis of high spatial resolution orbiter images during both the landing site selection process (Grant et al. 2018) and the subsequent strategic science assessment of the mission’s landing site in Jezero crater. Following the example set by the MSL Science Team before Curiosity’s landing, the Mars 2020 Science Team conducted a group mapping effort beginning one year before launch. The aim was to produce a detailed photogeologic map of the Perseverance rover landing ellipse and the surrounding area in and around western Jezero crater. This map was constructed to establish a common terminology and shared understanding within the Science Team of the geologic units present at the Perseverance field site, and to form the basis of scientific hypothesis development for strategic exploration, traverse planning, and sample caching for the Mars 2020 mission.

This paper presents the results of the Mars 2020 Science Team photogeologic mapping effort including a description of the methods by which the map was constructed, the criteria for distinguishing bedrock and surficial units, the integrated maps and unit descriptions, and several possible unit correlations that capture the current state of knowledge regarding the relative age relationships of major units in and around Jezero crater prior to Perseverance’s landing.

## Background

**Fig. 1:**
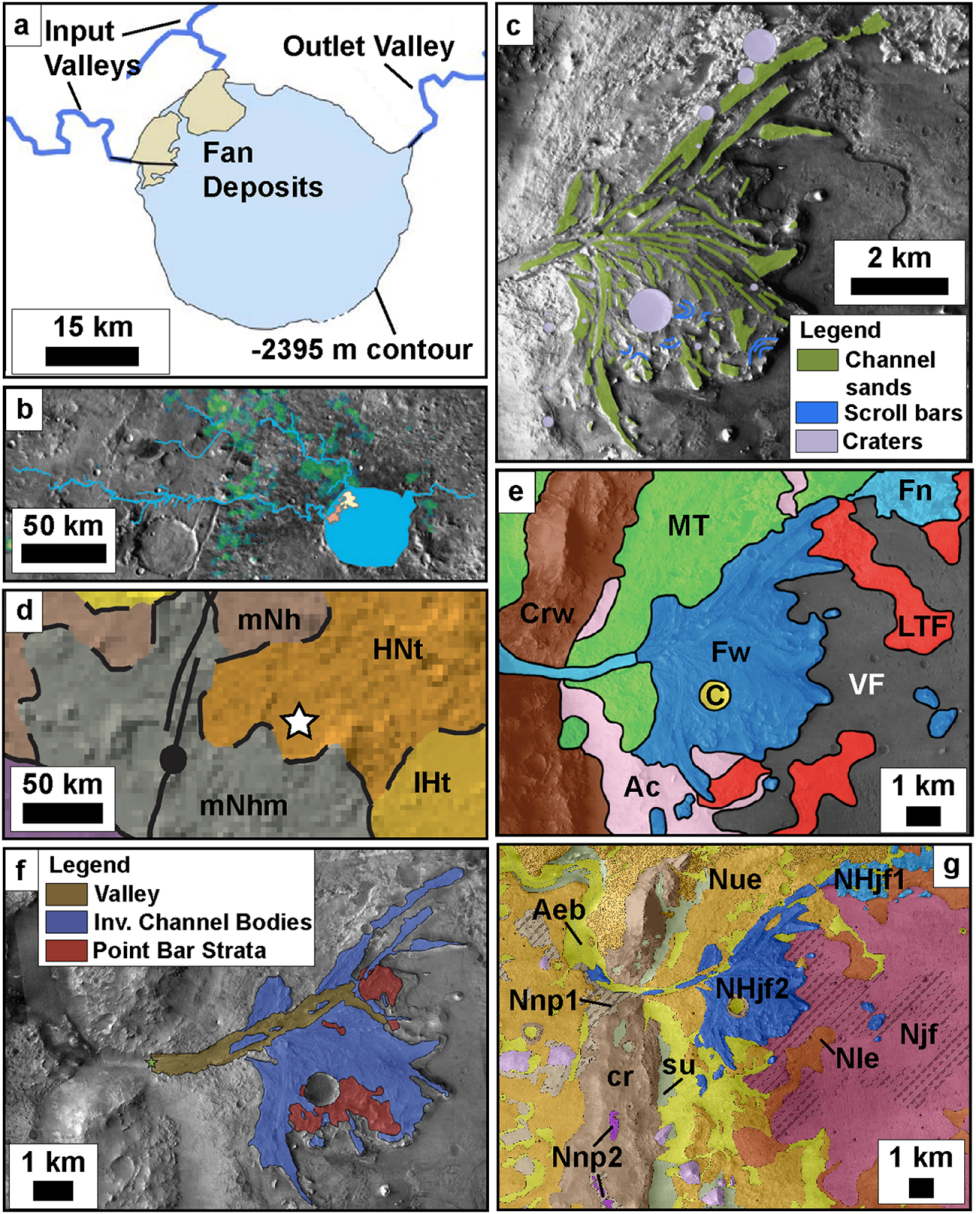
Previous mapping efforts in and around Jezero crater: (**a**) Inlet valleys, outlet valley, and western and northern fan deposits, modified from Fig. 1b in Fassett and Head (2005), (**b**) modified from Fig. 1c in Ehlmann et al. (2008); yellow is Northern delta, orange is Western delta, blue is channels and the extent of a lake if it were filled to the $-2395~\mbox{m}$ contour, (**c**) modified from Fig. 14b in Schon et al. (2012); channel sands, scroll bars, and craters of the western Jezero delta, (**d**) Jezero crater (white star) mapped in Tanaka et al. (2014); HNt is Hesperian and Noachian transition unit; mNhm is middle Noachian highland massif unit; lHt is late Hesperian transition unit; mNh is middle Noachian highland unit, (**e**) a portion of area mapped by Goudge et al. (2015) annotated with their map unit labels; MT is mottled terrain, Fn is northern fan deposit, Fw is western fan deposit, LTF is light-toned floor unit, VF is volcanic floor unit, Ac is surficial debris cover, C is impact crater, Crw is crater rim and wall material, (**f**) valleys, inverted channel bodies, and point bar strata modified from Fig. 2a in Goudge et al. (2018), (**g**) a portion of Jezero and the surrounding area mapped in Sun and Stack (2020). Nnp1 is Noachian Nili Planum 1, Nnp2 is Noachian Nili Planum 2, Nle is Noachian lower etched, Nue is Noachian upper etched, Njf is Noachian Jezero floor, NHjf1 and NHjf2 are Noachian Hesperian Jezero fan 1 and 2, respectively, cr is crater rim, su is smooth undivided, and Aeb is Amazonian eolian bedforms

Previously published geologic maps cover the area in and around Jezero crater at a variety of map scales, levels of detail, and areal extents. The first studies of Jezero crater (Fassett and Head 2005; Ehlmann et al. 2008) included simplified maps showing only the location and extent of the delta deposits within the crater (Fig. 1a and 1b, Table 1). Schon et al. (2012) constructed a more detailed, but partial, map of Jezero delta, showing the location of interpreted fluvio-deltaic channel bodies, scroll bar deposits, and several large craters (Fig. 1c, Table 1). The global United States Geological Survey (USGS) geologic map of Mars constructed at a 1:5,000,000 map scale (Tanaka et al. 2014) covered the entire crater and surrounding area, but depicted the units within Jezero and to the north as part of a single Hesperian to Noachian transition unit (HNt) and the terrains south of Jezero as a single middle Noachian highland massif unit (mNhm) (Fig. 1d, Table 1).

**Table 1:** Comparison of unit names between those used in this study and in previous mapping studies

This study	Fassett and Head (2005)	Ehlmann et al. (2008)	Schon et al. (2012)	Tanaka et al. (2014)	Goudge et al. (2015)	Goudge et al. (2018)	Sun and Stack (2020)
Aeolian bedforms, large	–	–	–	Hesperian and Noachian transition unit	Surficial debris cover	–	Eolian bedform unit
Aeolian bedforms, small	–	–	–	Hesperian and Noachian transition unit	Surficial debris cover	–	Eolian bedform unit
Undifferentiated smooth	–	–	–	Hesperian and Noachian transition unit	–	–	Smooth unit, undivided
Talus	–	–	–	Hesperian and Noachian transition unit	Surficial debris cover	–	–
Crater floor fractured 1	–	–	–	Hesperian and Noachian transition unit	Light-toned floor unit	–	Lower etched unit
Crater floor fractured 2	–	–	–	Hesperian and Noachian transition unit	Light-toned floor unit	–	Lower etched unit
Crater floor fractured rough	–	–	–	Hesperian and Noachian transition unit	Volcanic floor unit	–	Jezero floor unit
Margin fractured	–	–	–	Hesperian and Noachian transition unit	Mottled terrain	–	Upper etched unit
Delta blocky	Western fan	Western delta	Channel sands	Hesperian and Noachian transition unit	Western fan deposit	Inverted Channel Bodies	Jezero fan 2 unit
Delta thinly layered	Western fan	Western delta	–	Hesperian and Noachian transition unit	Western fan deposit	–	Jezero fan 2 unit
Delta thickly layered	Western fan	Western delta	–	Hesperian and Noachian transition unit	Western fan deposit	–	Jezero fan 2 unit
Delta truncated curvilinear layered	Western fan	Western delta	Scroll bars	Hesperian and Noachian transition unit	Western fan deposit	Point Bar Strata	Jezero fan 2 unit
Delta layered rough	Northern fan	Northern delta	–	Hesperian and Noachian transition unit	Northern fan deposit	–	Jezero fan 1 unit
Crater rim blocky	–	–	–	Hesperian and Noachian transition unit; middle Noachian highland massif unit	Crater rim and wall material	–	Crater rim unit
Crater rim breccia	–	–	–	Hesperian and Noachian transition unit; middle Noachian highland massif unit	Crater rim and wall material	–	Crater rim unit
Crater rim layered	–	–	–	Hesperian and Noachian transition unit; middle Noachian highland massif unit	Crater rim and wall material	–	Nili Planum 1 unit
Crater rim rough	–	–	–	middle Noachian highland massif unit	Thin dark capping unit	–	Nili Planum 2 unit
Neretva Vallis layered	Western input valley	Channels	–	Hesperian and Noachian transition unit	Valley networks	–	Jezero fan 2 unit
Nili Planum fractured	–	–	–	Hesperian and Noachian transition unit; middle Noachian highland massif unit	Mottled terrain; Eroded mottled terrain	–	Upper etched unit

Goudge et al. (2015) published the first complete geologic map of Jezero produced at a relatively large map scale (1:30,000) using a base map constructed of $\sim6~\mbox{m/pixel}$ MRO Context Camera (CTX; Malin et al. 2007) images (Fig. 1e, Table 1). Within Jezero crater, Goudge et al. (2015) identified several units exclusive to Jezero crater’s interior, as well as units interpreted to be stratigraphically equivalent to regionally extensive units mapped outside of Jezero crater. Goudge et al. (2015) identified a unit called the light-toned floor (LTF) to be the oldest exposed deposit to partially fill Jezero crater. They interpreted the LTF to be coeval with the mottled terrain (MT), a unit which was mapped around the inner margin of Jezero crater and outside the crater exposed over a substantial portion of the Jezero watershed. The LTF was identified on the basis of its light tone and prevalent fractures, while the MT was noted to have a mottled and degraded appearance. Both the LTF and MT exhibit olivine and carbonate signatures in visible-near-infrared spectroscopic data from the Compact Reconnaissance Imaging Spectrometer for Mars (CRISM; Murchie et al. 2007) instrument, though the presence and strength of the diagnostic mineral absorptions, especially for carbonate, are variable throughout these units (Mustard et al. 2009; Goudge et al. 2015; Brown et al. 2020; Horgan et al. 2020; Mandon et al. 2020). Mandon et al. (2020) estimated the emplacement age of the olivine-bearing unit throughout the Nili Fossae region (Goudge et al.’s 2015 MT unit) to be $3.82 \pm 0.07~\mbox{Ga}$.

Following the interpretation of Fassett and Head (2005) and Ehlmann et al. (2008), Goudge et al. (2015) distinguished two fan deposits, the western fan deposit (Fw) and the northern fan deposit (Fn), and interpreted both to have been emplaced after deposition of the LTF and MT units. Goudge et al. (2015) mapped the western fan as a single unit, but Goudge et al. (2018) distinguished additional detail within this fan by mapping out the inlet valley, inverted channel bodies, and point bar strata, although portions of the delta and the adjacent units remained unmapped (Fig. 1f, Table 1). Fe/Mg smectite and carbonate have been detected within both the northern and western fan deposits, as have mafic minerals such as olivine and low-calcium pyroxene (Goudge et al. 2015; Horgan et al. 2020).

The youngest bedrock unit mapped by Goudge et al. (2015) within Jezero was called the volcanic floor unit (VF). They described the VF as a smooth, crater-retaining, and relatively thin unit ($<10~\mbox{m}$ thick) spanning much of the Jezero crater floor. On the basis of its apparent dark tone, near-infrared spectroscopic detection of mafic rock-forming minerals (olivine and pyroxene), interpreted embayment of the fan deposits, and erosional resistance as expressed by small impact crater retention, Goudge et al. (2015) interpreted the VF to be a lava despite acknowledging that they found no evidence for an associated vent or volcanic edifice. Goudge et al. (2012) used crater counting methods to determine an emplacement age for the VF of approximately $3.45_{-0.67}^{+0.12}~\mbox{Ga}$, although a younger age of $\sim1.4~\mbox{Ga}$ (Schon et al. 2012) was also derived for this unit. A more recent study by Shahrzad et al. (2019) discussed this discrepancy and presented an age of $2.6\pm0.5~\mbox{Ga}$ for the crater floor. Goudge et al. (2015) also mapped a relatively thin, crater-retaining, and mesa-forming unit called the thin, dark capping unit (Tcu) of unknown origin and relative age on Jezero’s western crater rim.

At the time of writing, a USGS Scientific Investigations 1:75,000 scale map of the Jezero and Nili Planum region is in press (Sun and Stack 2020) (Fig. 1f, Table 1). Unit distinctions of this more recent effort appear similar to that of Goudge et al. (2015), but the Sun and Stack (2020) map extends continuous coverage to Nili Planum east and south of Jezero crater.

## Data and Methods

**Fig. 2:**
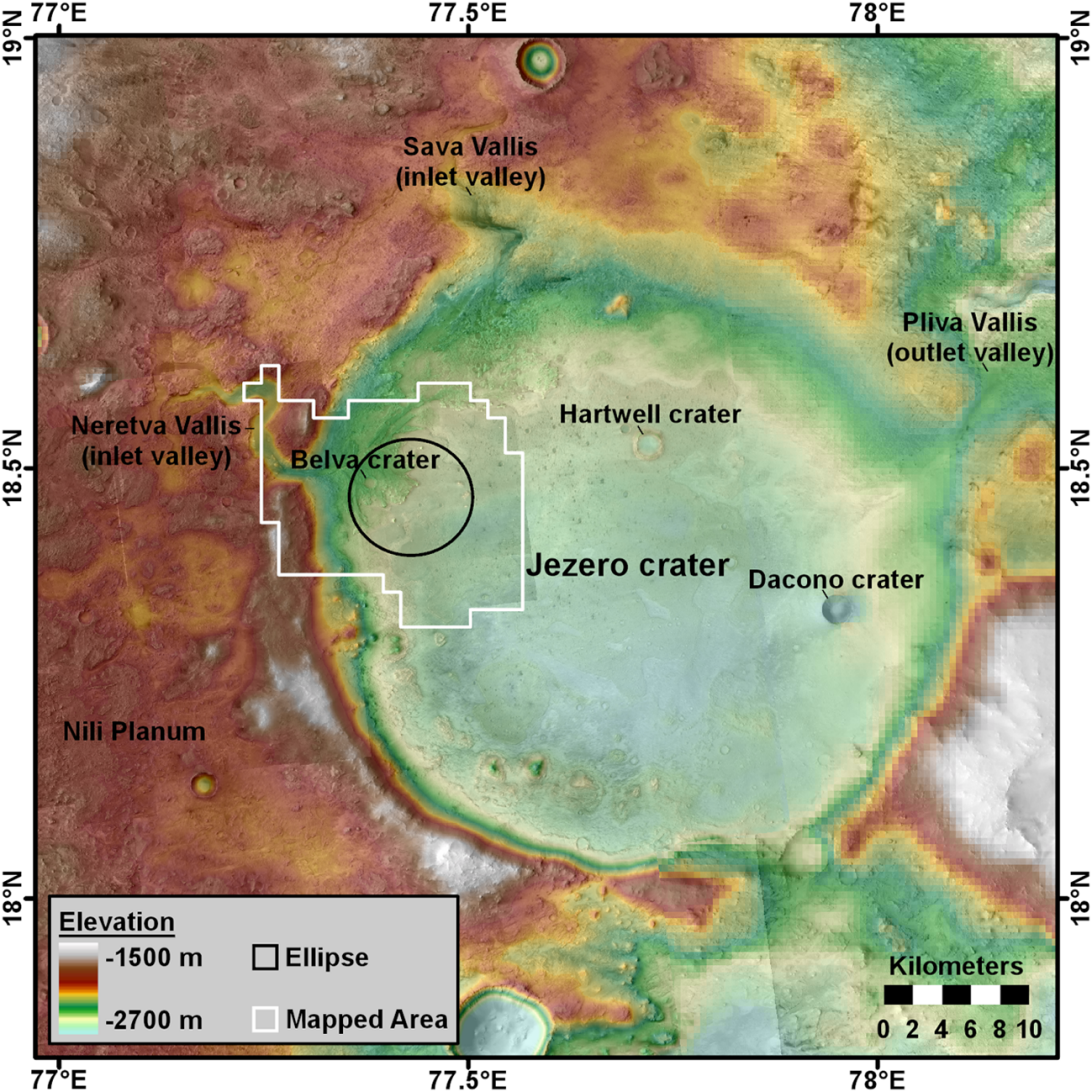
Map of Jezero crater and Nili Planum showing the Mars 2020 landing ellipse in black and this study’s map area outlined in white. Colors correspond to topography from HiRISE and CTX digital terrain models and from the MOLA overlain on CTX and HiRISE image basemaps

The Mars 2020 Science Team map of Jezero crater was constructed using a 25 cm/pixel visible image base map consisting of HiRISE red filter images listed in Online Resource 1. This base map, which was originally constructed to evaluate the safety of the Jezero landing site for hardware entry, descent, and landing (Fergason et al. 2020), dictated the extent of the Science Team’s mapping effort (Fig. 2). A digital terrain model constructed from HiRISE stereo image pairs with different viewing geometries was used to provide a three dimensional perspective on outcrop exposures and to help correct for image distortions that resulted from perspective tilting and terrain effects (Fergason et al. 2020). The HiRISE mosaic was tied to an MRO CTX 6 m/pixel orthoimage mosaic (Fergason et al. 2019), which itself had been co-registered to High Resolution Stereo Camera (HRSC; Jaumann et al. 2007) 12.5 m/pixel images and Mars Orbiter Laser Altimeter (MOLA; Smith et al. 2001) topographic products to provide a geographic tie to the martian elevation datum and the International Astronomical Union (IAU) Mars coordinate system (Seidelmann et al. 2002). Mapping was performed primarily using the HiRISE visible image base map, but also used were: the HiRISE-derived digital terrain model, a slope map, stereo anaglyphs, an artificial hillshade, a colorized shaded relief map, and HiRISE-plus MOLA-derived topographic contours at 1, 5, 10, 20, 50, and 100 meter intervals. The team also used a CRISM false color map (Seelos et al. 2013; Online Resource 1).

**Fig. 3:**
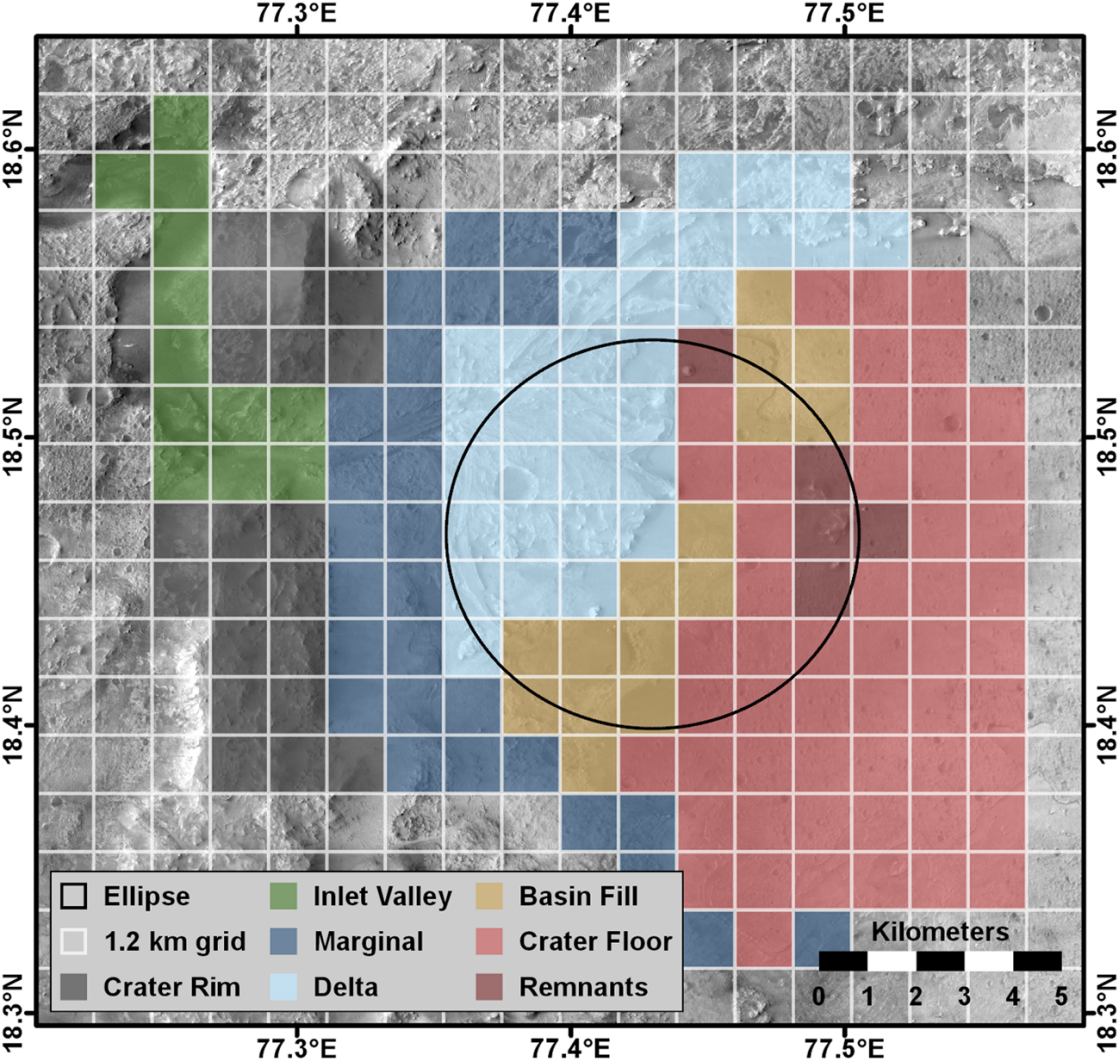
Map of the 1.2 km by 1.2 km quadrangles mapped by the Mars 2020 Science Team color-coded by geographic areas that correspond to the team’s mapping groups. The extent of this map corresponds to the area of greatest scientific interest to the Mars 2020 Science Team and where high-resolution HiRISE image data were available

A grid of 1.2 km by 1.2 km quadrangles (“quads”), each informally named after an Earth-based national park or preserve (Online Resource 2), was overlain on the region of available orbital data (Fig. 3). The 166 quads with HiRISE image coverage were then subdivided by geographic setting: crater floor, basin fill, delta, marginal deposits, crater rim, and inlet valley (Fig. 3). Two to three “Mapping Leads” from the Mars 2020 Science Team were designated for each quad grouping, and quads were assigned to 63 individual Science Team member volunteers. Mapping Leads facilitated discussion amongst their group’s quad mappers to establish preliminary unit identification and reconciliation prior to mapping to ensure consistency across quad boundaries.

The team mapping effort was carried out in three phases: Phase 1 (May-July 2019), Phase 2 (July-September 2019), and Phase 3 (September 2019-April 2020). Phase 1 involved the assignment of quads to Science Team members, tutorials and training sessions with the mapping tools, and initial unit identification and discussion within each group. Phase 2 consisted primarily of quad mapping and biweekly Science Team discussions at which each sub-group presented progress reports and new findings. Phase 2 concluded with completion of individual quad maps and unit descriptions. Phase 3 involved compiling the quads to form a unified map in which unit boundaries were completely reconciled across quad borders and between mapping groups. This effort included iteration with the Mapping Leads and discussions with the Science Team to reach consensus geologic interpretations supported by the photogeologic map.

**Fig. 4:**
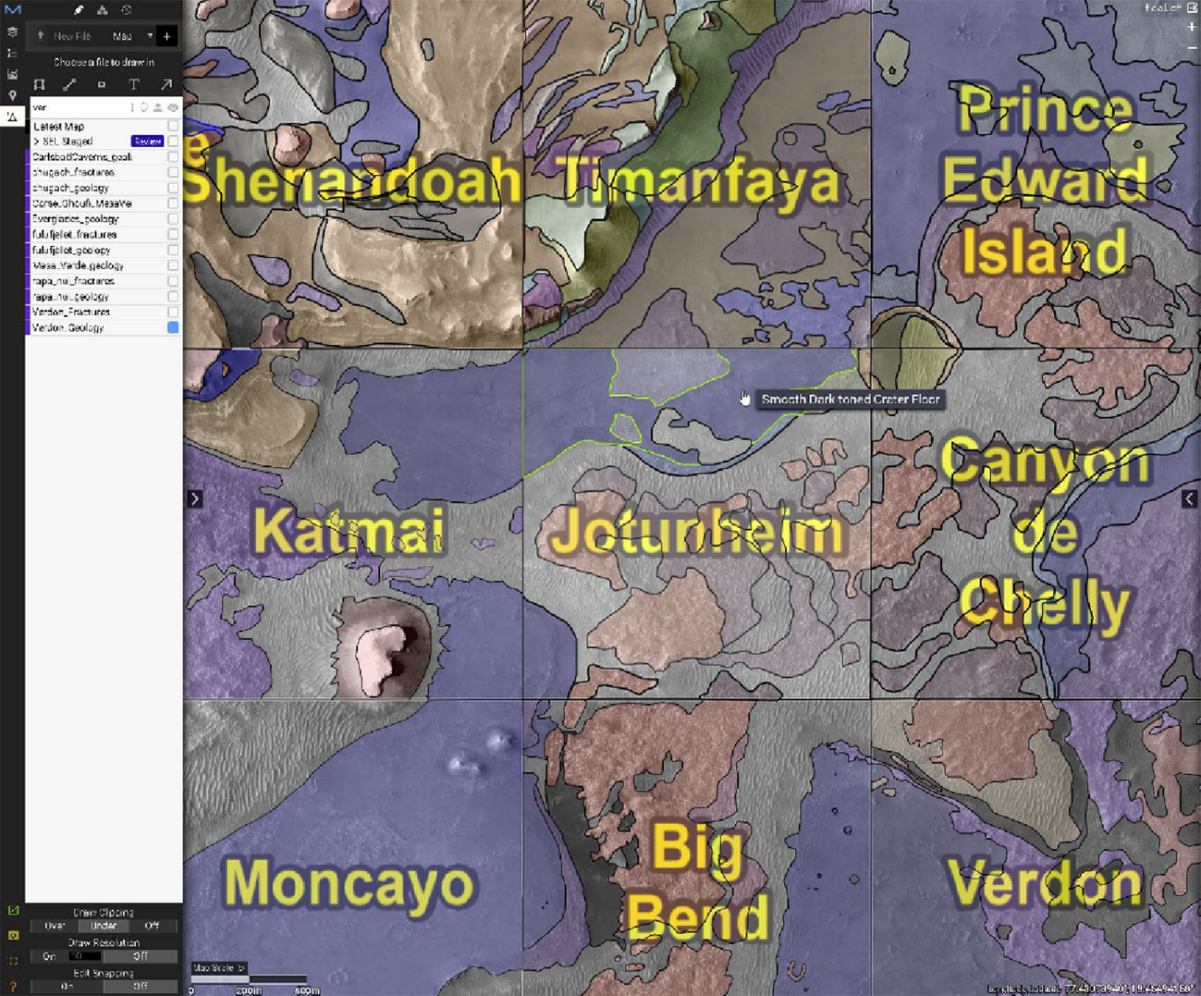
The CAMP tool developed by Calef and Soliman (2019) and used by the Mars 2020 Science Team to construct the photogeologic map. 1.2 by 1.2 km quadrangles were displayed in CAMP and assigned to individual team members who mapped units within the tool. Mapped geologic units shown are the raw, uncorrelated boundaries by mapping quad

The mapping effort was conducted using the CAMP (Campaign Analysis Mapping and Planning) tool, part of the MMGIS (MultiMission Geographic Information System) open source software package funded, developed, and maintained by the NASA AMMOS (Advanced Multi-Mission Operations System) (Calef and Soliman 2019) (Fig. 4). The software is part of a web-based spatial data infrastructure that supports a dispersed, international team working on science operations for planetary missions. MMGIS is a multi-view, web-based mapping package that provides two-dimensional and three-dimensional views of spatial data. This software stores all vector layers in PostgreSQL (version 9.6) with the POSTGIS extension (version 2) as a spatially enabled database. Individual raster and vector layers can be turned on/off, queried for their raw values (e.g., elevation), or measured with built-in tools. For this mapping effort, CAMP provided a web-based, two-dimensional map view in a “web Mercator” projection onto which individual geologic unit vector layers could be digitized.

The HiRISE mosaic base map and supplementary datasets were imported into CAMP and individual science team members established a vector geologic mapping layer based on their assigned quad(s). Each layer was digitized as a series of polygons at a map scale of 1:5000. Units were distinguished if they exhibited a distinct texture, tone, color, or topographic expression. In several cases, units were distinguished by elevation range and/or geographic setting, e.g., inside versus outside Jezero crater. In addition to exposed bedrock units, surficial units were also recognized throughout the mapping area. Surficial units were defined as those that likely do not extend or project into the subsurface, but rather obscure or partly obscure the bedrock substrate. Surficial units were mapped as distinct units if they covered the underlying bedrock over areas discernible at map scale, even if the cover was inferred to be relatively thin. Areas with partial cover for which differentiating bedrock from surficial deposit at map scale was challenging were also recognized. Areas mapped as “minor cover” include $>0$ to $\sim25\%$ cover; areas mapped as “moderate cover” include $\sim25\mbox{--}75\%$ cover. For each mapped bedrock or surficial unit, the mapping team characterized and described the distinguishing criteria and provided a type location (Table 2). Once units from each mappers’ quadrangles were digitized, quad maps were merged, edited, and finalized into a single map file using Esri’s ArcGIS Pro 2.3 software (Online Resource 3).

**Table 2:** Summary of mapped surficial and bedrock units

Group	Unit name (this study)	Unit abbreviation	Unit name (Goudge et al. 2015)	Unit description	Interpretation	Type location(s) (lat/lon)
Surficial units	Aeolian bedforms, large	Ab-l	Surficial debris cover	Light-toned, parallel, and straight-crested bedforms ∼10s to 100s of meters in length and ∼1–10s of meters in wavelength	Transverse aeolian ridges	77.379, 18.483 and 77.337, 18.433
	Aeolian bedforms, small	Ab-s	Surficial debris cover	Dark-toned, sub-parallel, straight-crested bedforms ∼1–10s of meters in length and 1 to several meters in wavelength. Reticulate patterns are common	Aeolian wind ripples	77.421, 18.427
	Undifferentiated smooth	Us	–	Widespread smooth, dark-toned deposits that drape topography	Unconsolidated mantling deposits variably composed of dust, sand, pebbles, cobbles	77.431, 18.402
	Talus	T	Surficial debris cover	Boulder accumulations on slopes and below eroded outcrops	Blocks eroded from the bedrock via physical weathering	77.429, 18.497
Jezero crater floor	Crater floor fractured 1	Cf-f-1	Light-toned floor unit	Massive, light-toned fractured and blocky bedrock exposed on the crater floor below $-2530~\mbox{m}$ elevation	Unspecific tephra, airfall, aeolian, or lacustrine deposit	77.413, 18.410
	Crater floor fractured 2	Cf-f-2	Light-toned floor unit	Light-toned, rough, and fractured bedrock that crops out between −2530 and $-2440~\mbox{m}$ elevation	Unspecified tephra, airfall, aeolian, or lacustrine deposit	77.447, 18.560
	Crater floor fractured rough	Cf-fr	Volcanic floor unit	Light- to medium-toned, rough, boulder-producing unit that is highly crater-retaining. Polygonal fracture networks are common	Unspecified tephra, airfall, aeolian, or lacustrine deposit	77.467, 18.340
Jezero crater margin	Margin fractured	M-f	Mottled terrain	Light-toned fractured bedrock inside Jezero crater between the elevation ranges of $-2440~\mbox{m}$ and $-2190~\mbox{m}$ south of the Neretva Vallis and $-2440~\mbox{m}$ and $-2240~\mbox{m}$ north of Neretva Vallis. Forms a low relief, less blocky expression, and blocky, ridge-forming outcrops	Unspecified tephra or marginal lacustrine deposit	77.335, 18.476
Jezero crater delta	Delta blocky	D-bl	Western fan deposit	Intermediate-toned blocky deposit that forms steep-sided, boulder-shedding elongate ridges on the delta’s upper surface	Coarse-grained fluvial channel deposits	77.385, 18.501
	Delta thinly layered	D-tnl	Western fan deposit	Stratified sequence of $<1~\mbox{m}$ thick alternating light and dark planar bands. Locally deformed	Fine-grained prodelta or distal lacustrine deposits	77.350, 18.451
	Delta thickly layered	D-tkl	Western fan deposit	Light-toned, resistant strata up to several meters thick	Channel lobes at the toe of the delta slope or delta plain alluvial or floodplain deposits	77.417, 18.524
	Delta truncated curvilinear layered	D-tcl	Western fan deposit	Curvilinear, decimeter-scale sets of alternating light- and dark-toned layers that truncate against one another over length-scales of tens of meters	Laterally accreting point bars, or subaqueous channel-levee complexes	77.387, 18.472
	Delta layered rough	D-lr	Northern fan deposit	Light-toned, parallel m-thick stratified deposit northeast of the western delta	Distal deltaic deposits	77.481, 18.583
Jezero crater rim and beyond	Crater rim blocky	Cr-bl	Crater rim and wall material	Intermediate-toned unit that forms resistant high-standing ridges that erode into boulders	Pre-Jezero/impact basement bedrock of unspecified sedimentary or volcanic origin	77.292, 18.467
	Crater rim breccia	Cr-br	Crater rim and wall material	Brecciated and disrupted light- and intermediate-toned bedrock exposed on the Nili Planum-facing slope of the Jezero crater rim	Syn-Isidis or syn-Jezero impact breccia	77.269, 18.445
	Crater rim layered	Cr-l	Crater rim and wall material	Light-toned stratified and polygonally fractured unit that is occasionally faulted and disrupted	Pre-Jezero/impact basement bedrock of unspecified sedimentary or volcanic origin	77.260, 18.459
	Crater rim rough	Cr-r	Pitted capping unit	Light-toned unit characterized by high crater retention, a rough texture, and polygonal fractures	Unspecified clastic sedimentary or explosive volcanic deposit	77.275, 18.386
	Neretva Vallis layered	NV-l	–	Light- to intermediate-toned layered outcrops exhibiting m-scale polygonal fractures	Fluvial deposits	77.256, 18.509
	Nili Planum fractured	NP-f	Mottled terrain; Eroded mottled terrain	Light-toned fractured outcrop occurring above $-2240~\mbox{m}$ elevation north of Neretva Vallis and above $-2190~\mbox{m}$ elevation south of Neretva Vallis	Unspecified tephra, airfall, or aeolian deposit	77.274, 18.537

**Fig. 5:**
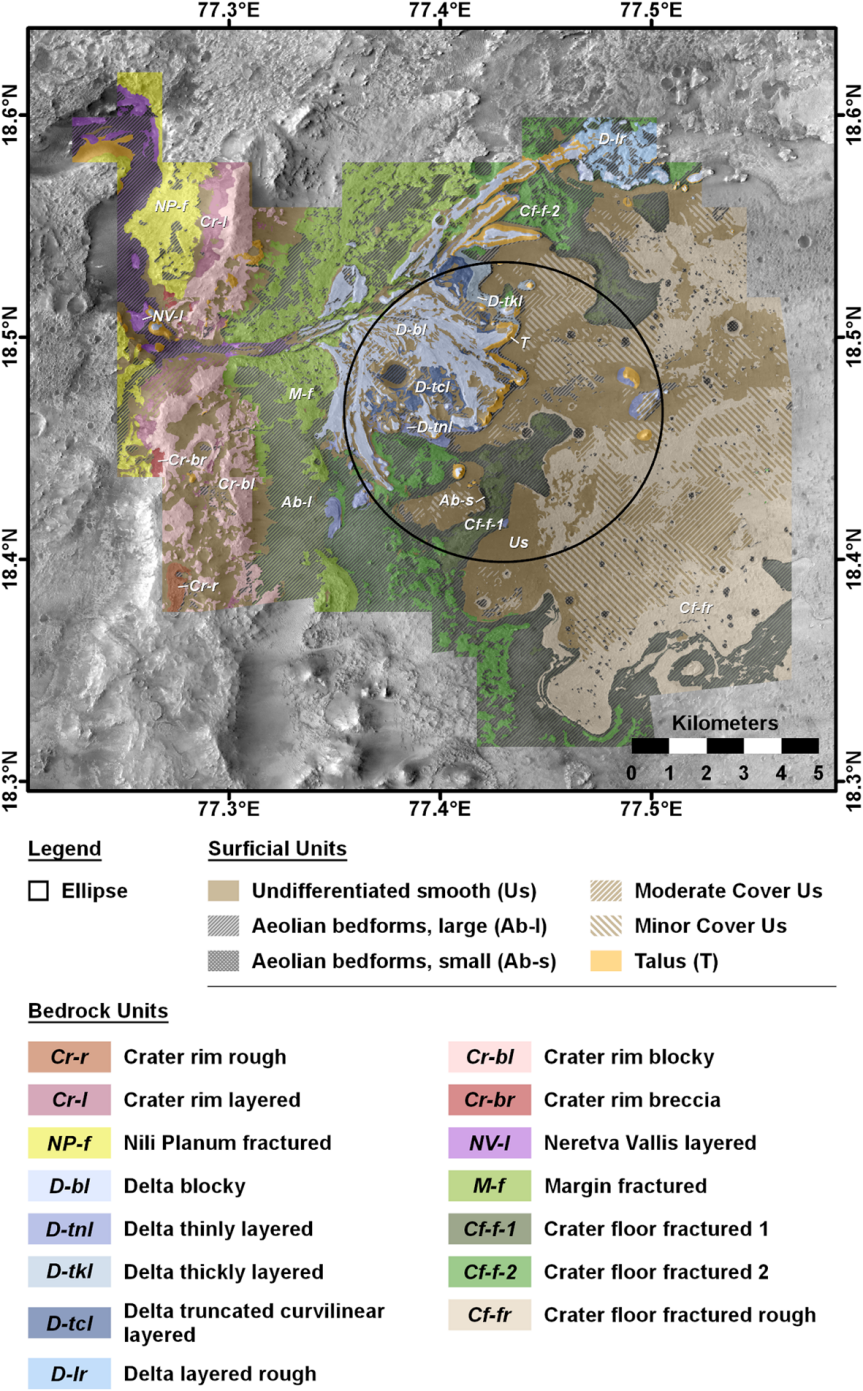
Integrated surface exposure photogeologic map showing bedrock and surficial units mapped by the Mars 2020 Science Team in and around the Perseverance landing site in Jezero crater

**Fig. 6:**
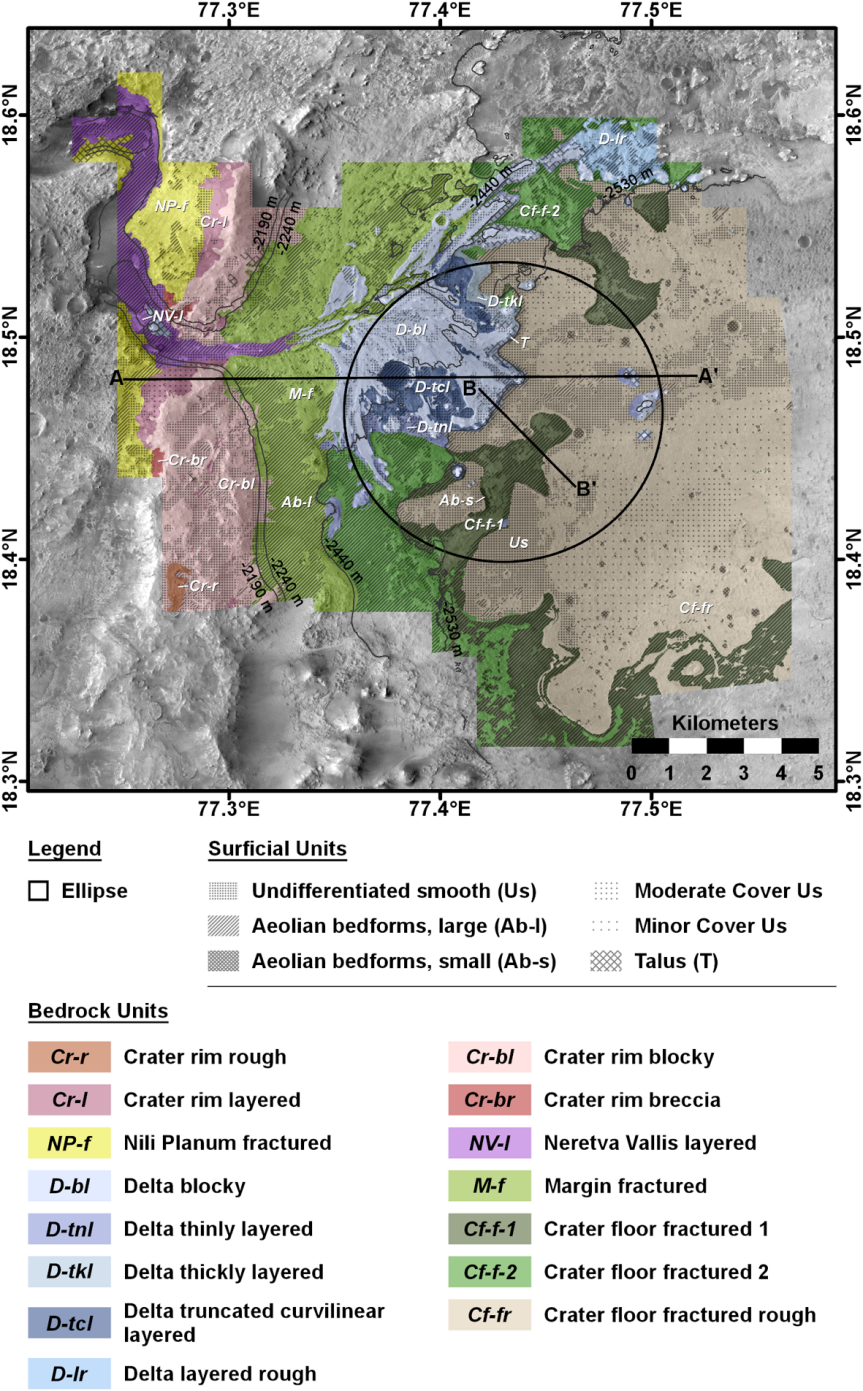
Photogeologic map emphasizing bedrock units within the mapped area. Transects A to A’ and B to B’ represent the location of cross-sections shown in Figs. 14 through 17

The surface exposure map, which shows the distribution of both bedrock units and surficial deposits mapped at the present-day surface, is displayed in Fig. 5. Partial covering of bedrock units by surficial units is illustrated throughout the map area with colored hatched overlays. This map, in addition to showing the location of bedrock exposures, highlights the extent of surficial deposits, including aeolian bedforms, throughout the study area. The map in Fig. 6 emphasizes the distribution of inferred bedrock units with all surficial units displayed as simple hatched or stippled patterns. For areas in which the present-day surface is completely obscured by surficial units, the underlying bedrock geology was inferred based on the surrounding outcrop. This map was used to construct cross-sections illustrating possible unit correlations along two topographic transects, A to A’ and B to B’ (Fig. 6). Cross-section A to A’ was selected to show unit relationships inside and outside the crater; cross-section B to B’ was selected to highlight the relationship between the western delta and the units that comprise the Jezero crater floor inside Perseverance’s landing ellipse.

## Unit Descriptions and Interpretations

Four surficial units and fifteen distinct bedrock units were distinguished in the map area (Figs. 5 and 6). The surficial units include two that consist of aeolian bedforms, an undifferentiated smooth (at map scale) unit interpreted to mantle overlying bedrock throughout the map area, and talus. Four bedrock units are exposed on the crater rim, and a layered unit crops out within the walls and floor of Neretva Vallis, the western inlet valley. Fractured, commonly light-toned units are located both inside the Jezero and on Nili Planum beyond the crater rim. These units are morphologically similar, but have been distinguished as separate units primarily based on elevation contours that coincide with changes in the geographic setting of the deposits, i.e., crater floor, Jezero interior margin, or outside the crater on Nili Planum. Three fractured units are exposed on the Jezero crater floor and a fourth is defined along the interior margin of the crater rim.

**Fig. 7:**
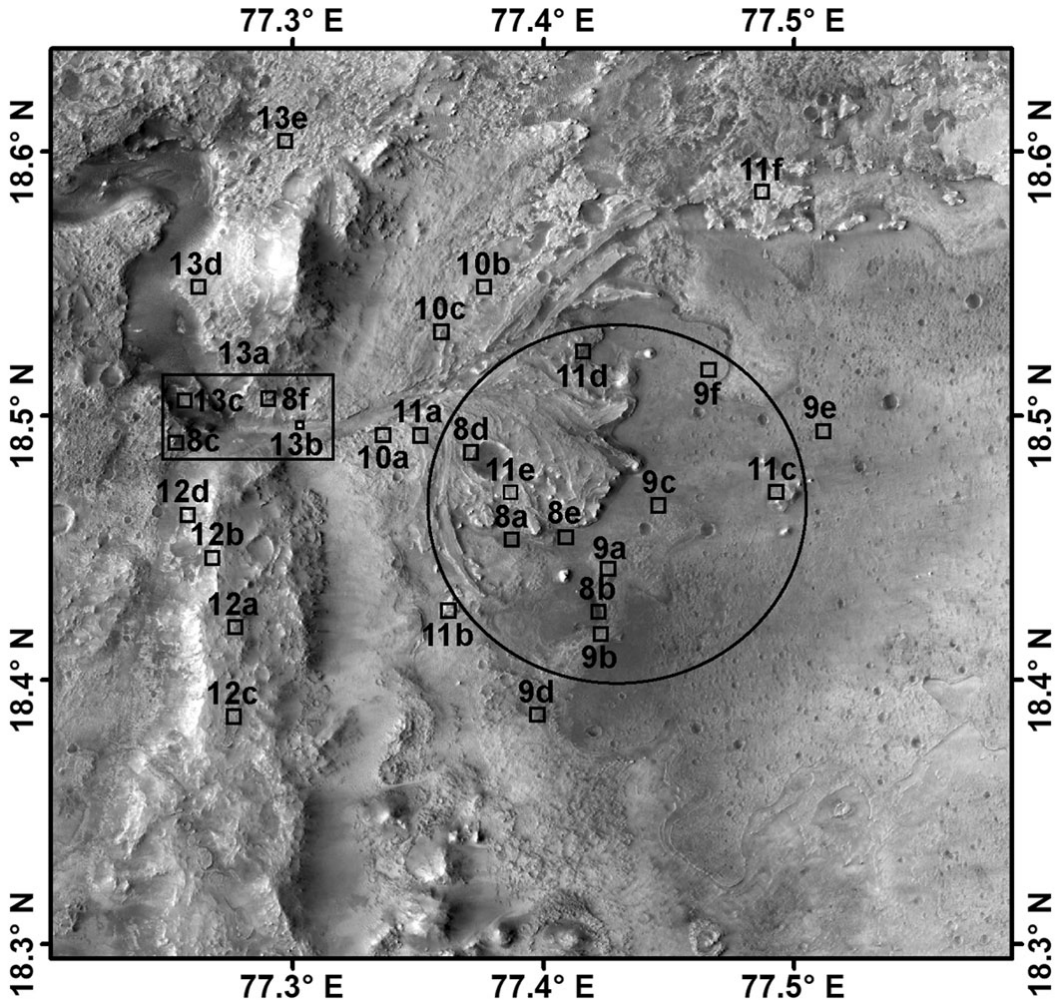
Locations of unit examples displayed in Figs. 8–13

**Fig. 8:**
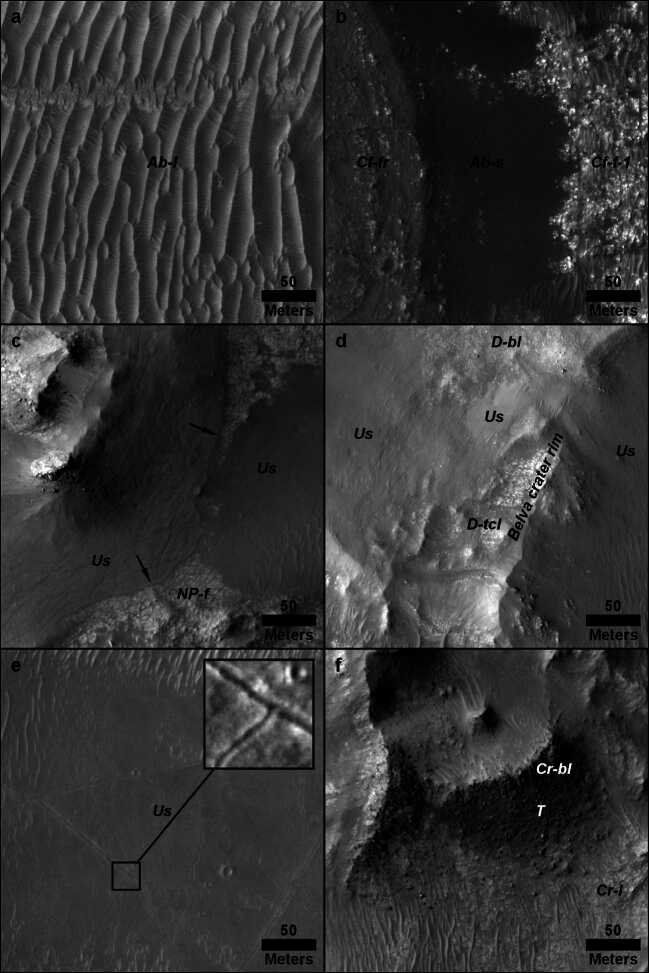
Surficial units observed in and around Jezero crater: (**a**) large aeolian bedforms (Ab-l), (**b**) small aeolian bedform (Ab-s), (**c**) undifferentiated smooth unit (Us) in sharp contact (black arrows) with adjacent, underlying bedrock (NP-f), (**d**) Us inside, outside, and draping the Belva crater rim on the top surface of the Jezero delta, (**e**) Us on the crater floor showing fracture networks $\sim100\mbox{s}$ of meters in length (inset, with enhanced contrast), (**f**) talus (T) on the Jezero crater rim

**Fig. 9:**
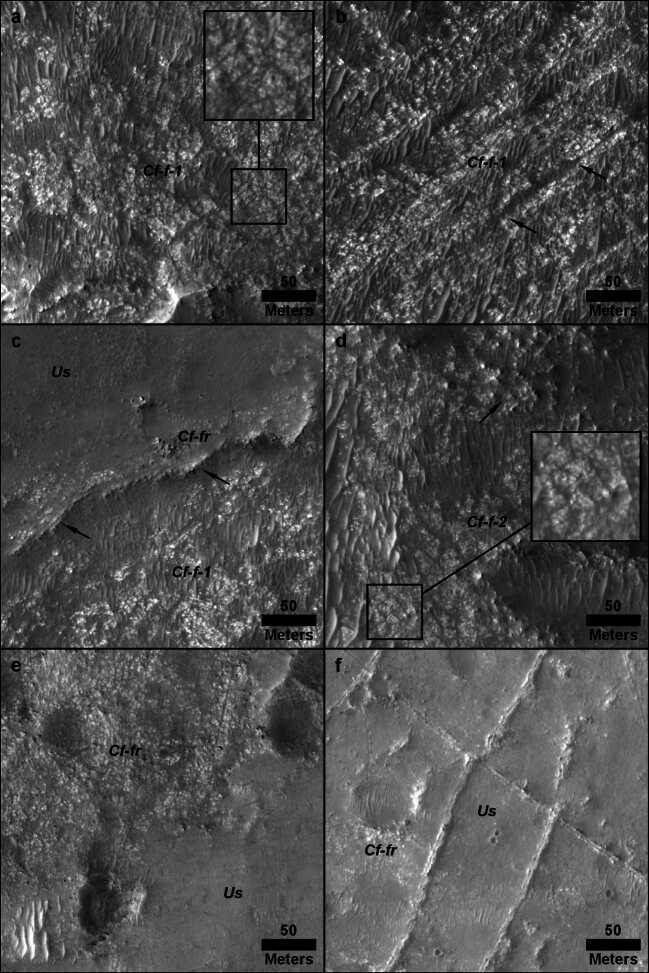
Examples of fractured and fractured rough units on the Jezero crater floor: (**a**) crater floor fractured 1 (Cf-f-1) with inset showing polygonal fractures, (**b**) crater floor fractured 1 (Cf-f-1) unit showing northeast-southwest trending furrows spaced $\sim50\mbox{--}75~\mbox{m}$ apart, (**c**) Topographic step (black arrows) that forms the contact between Cf-f-1 and adjacent crater floor fractured rough (Cf-fr) unit, (**d**) polygonal fractures (inset) and pock-marked texture (black arrows) of the crater floor fractured 2 (Cf-f-2) unit, (**e**) exposure of Cf-fr with little to no overlying undifferentiated smooth unit (Us) adjacent to area covered by Us, (**f**) Cf-fr displaying raised fractures and “moderate” coverage by Us

**Fig. 10:**
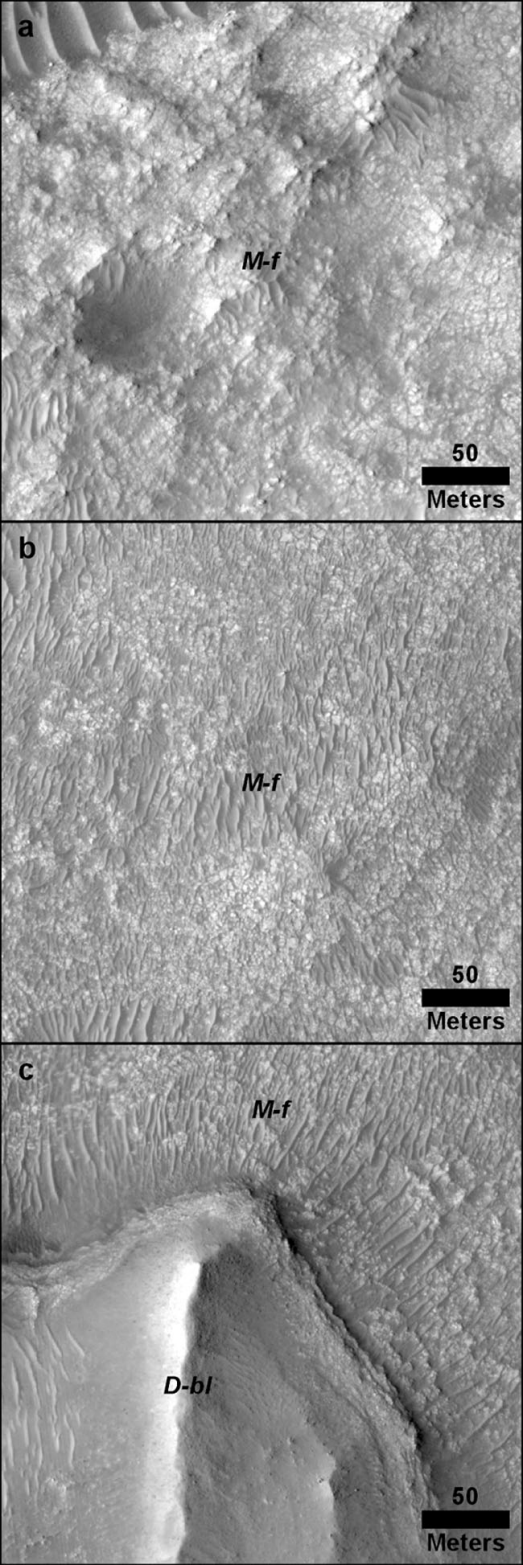
Examples of the margin fractured (M-f) unit: (**a**) blocky expression of the M-f, (**b**) low-relief expression of the M-f, (**c**) delta blocky (D-bl) unit overlying the M-f

**Fig. 11:**
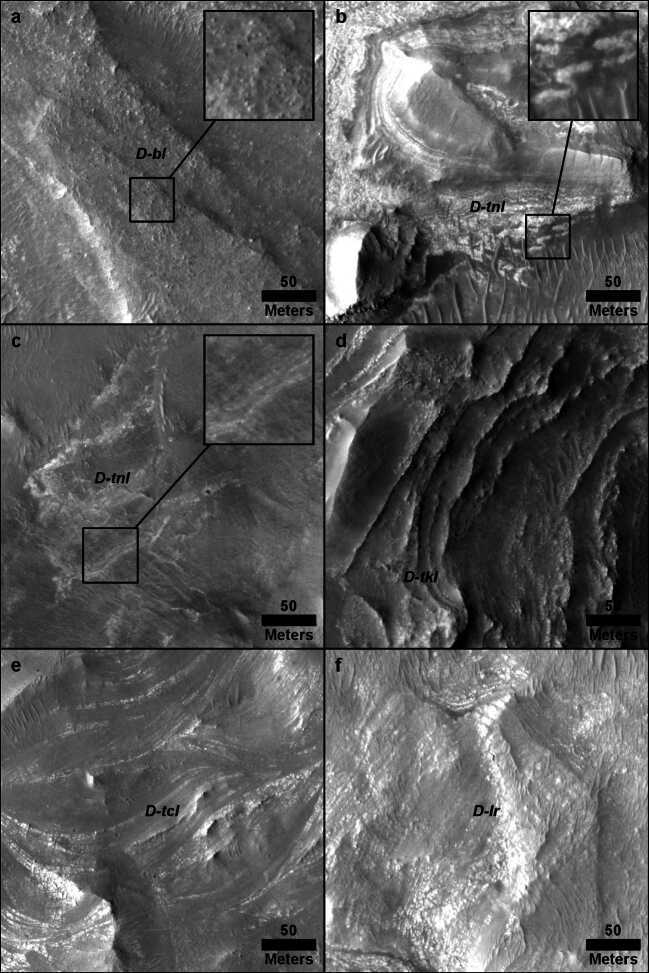
Representative examples of the Jezero delta units: (**a**) delta blocky (D-bl) unit; inset shows individual blocks on the upper surface of the delta, (**b**) delta thinly layered (D-tnl) unit; inset highlights contorted layers, (**c**) delta thinly layered (D-tnl) unit exposed at the base of a remnant mound east of the Jezero delta; inset highlights layers within the remnant mound, (**d**) delta thickly layered (D-tkl) unit. (**e**) delta truncated curvilinear layered (D-tcl) unit, (**f**) delta layered rough (D-lr) unit comprising the fan deposit northeast of, and adjacent to, the Jezero delta

**Fig. 12:**
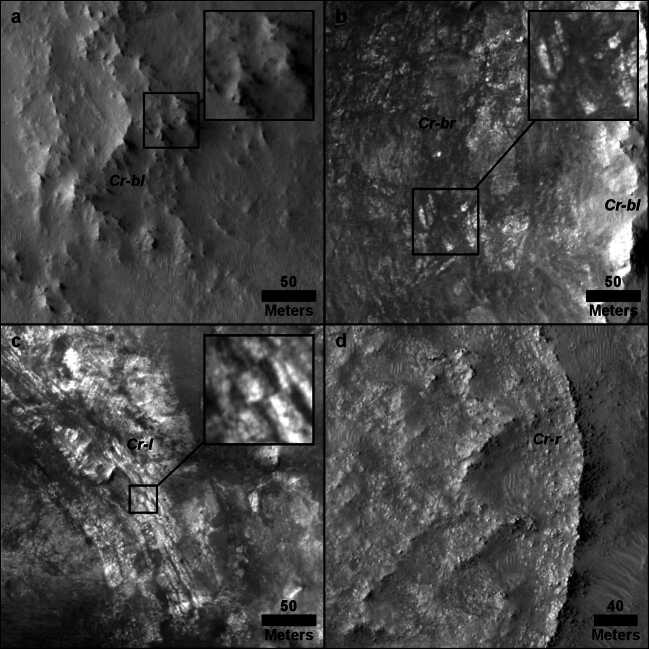
Units on the Jezero crater rim: (**a**) crater rim blocky unit (CR-bl), (**b**) crater rim breccia (Cr-br); inset shows individual light-toned blocks, (**c**) crater rim layered (Cr-l) unit; inset shows faulting within the Cr-l unit, (**d**) crater rim rough (Cr-r) unit

**Fig. 13:**
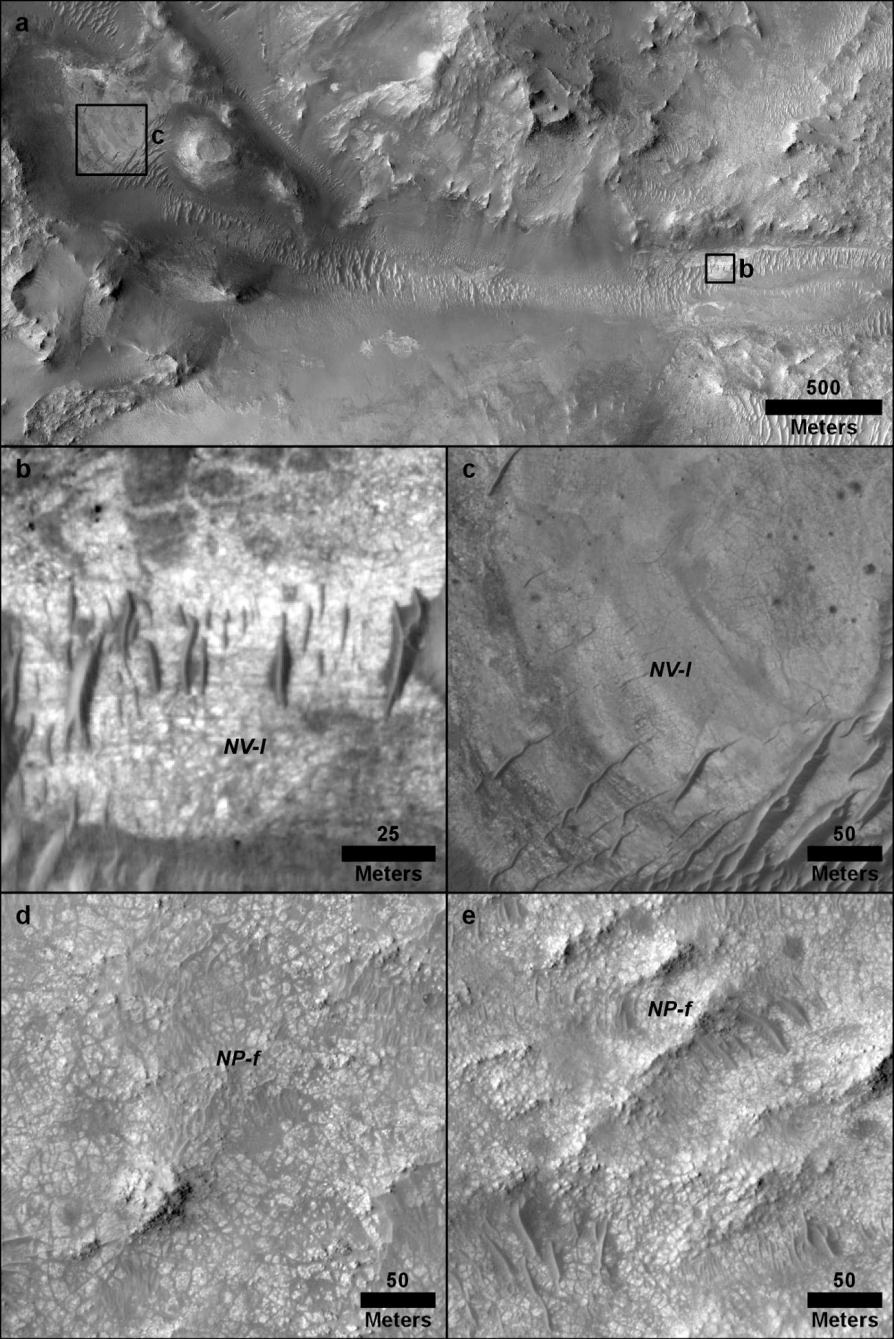
Units within Neretva Vallis and on Nili Planum: (**a**) Occurrences of Neretva Vallis layered (NV-l) unit within Neretva Vallis shown in (**b**) and (**c**), (**b**) exposure of NV-l just inside the rim of Jezero crater, (**c**) another exposure of NV-l within Neretva Vallis outside of Jezero crater, (**d**) low-relief expression of the Nili Planum fractured (NP-f) unit, (**e**) blocky, ridged expression of the NP-f

Five distinct bedrock units were recognized within the Jezero delta deposits. These include the layered rough unit that makes up the majority of the fan deposit northeast of and adjacent to the western delta, three layered units observed within the western delta that exhibit distinct layered morphologies and/or geometries, and a blocky unit that comprises much of the upper surface of the western delta. Figure 7 shows the location of outcrop examples described in the text. These representative outcrops are displayed at map scale in Figs. 8–13.

### Surficial Units

#### Aeolian Bedforms, Large (Ab-l)

Large aeolian bedforms were mapped over areas within which light to intermediate-toned bedforms cover approximately 80% or more of the surface area, and where the underlying substrate cannot be clearly differentiated or identified at map scale (Fig. 8a). The bedforms, which are commonly light-toned at their crests and dark within the troughs, are generally straight-crested and most commonly trend approximately north-south. These bedforms vary in length from $\sim10\mbox{s}$ to several 100s of meters, display individual widths on the order of $<1$ to $\sim10~\mbox{m}$, and wavelengths commonly on the order of several meters to 10s of meters. Bedform amplitude is on the order of several meters or less, but is only resolved among the taller examples via the HiRISE-derived digital terrain model. Bifurcations are common, but the crestlines of all the largest bedforms are generally parallel to sub-parallel. Craters are not observed on the bedforms suggesting both a relatively young age for the bedforms compared to the cratered bedrock units and a composition of unconsolidated sediment.

Large aeolian bedforms occur throughout the study area, but are most commonly observed in local topographic lows such as impact craters and at the bases of steep slopes. Bedforms are most pervasive inside Jezero in a low-relief area between the crater rim and the rock units of the crater floor. These bedforms are interpreted to be transverse aeolian ridges (TARs) (Day and Dorn 2019), which are light-toned, symmetrical bedforms oriented orthogonal to the dominant wind direction (e.g., Zimbelman 2010). Given their consistent N-S orientation and accumulation on the western side of the crater, the TARs in Jezero suggest a dominant easterly wind regime (Day and Dorn 2019). Gradational transitions between the large aeolian bedforms and more complex secondary bedform patterns throughout the map area indicate multiple, variable wind directions within Jezero crater, perhaps influenced by local topography.

#### Aeolian Bedforms, Small (Ab-s)

Dark, sub-parallel, straight-crested bedforms oriented predominantly N-S occur throughout the map area within local topographic lows such as crater interiors and at the bases of steep slopes (Fig. 8b). Bedforms are up to a few 10s of meters in length and exhibit wavelengths of $\sim3~\mbox{m}$. Bedform amplitude is too small to be resolved in the digital terrain model, but assuming the ripples have shallow slopes below the angle of repose ($\sim30^{\circ}$), the amplitude is likely on the order of several 10s of cm at most. Reticulate and polygonal patterns are common, indicating bimodal and multimodal wind directions. These bedforms are relatively uncommon within the study area compared to the large aeolian bedforms (Ab-l, Fig. 8a), which are distributed throughout the map area. That the small aeolian bedforms do not preserve small impact craters and appear to be relatively dust-free given their dark tone supports a relatively young age and an inference that they consist of unconsolidated sediment. Given the scale, morphology, low albedo, and setting of the bedforms, they are interpreted to be recently active aeolian ripples.

#### Undifferentiated Smooth Unit (Us)

The undifferentiated smooth unit is the designation used for any deposit within the map area that has a medium to dark uniform tone and generally lacks resolvable texture at map scale (Fig. 8c, 8d, and 8e). No stratification is observed within deposits of this unit, but exposures do, in some cases, exhibit minor light and dark mottling and subtle lineation. Deposits mapped as undifferentiated smooth unit appear to conform to topography, often occurring within impact craters and on slopes (Fig. 8d). These deposits occur across the map area and over nearly the full range of elevations observed within the study area. Undifferentiated smooth deposits are observed to overlie bedrock units exposed on the Jezero crater floor, within and on the delta, on deposits exposed along the inner margin of Jezero, and on the crater rim. Exposures vary in size, but continuous expanses up to several square kilometers are observed, particularly on the Jezero crater floor and crater rim. Deposits mapped as undifferentiated smooth unit most commonly exhibit gradational transitions to nearby units, particularly when adjacent to aeolian bedforms. However, in some places, sharply defined boundaries occur between the undifferentiated smooth unit and subjacent bedrock units (Fig. 8c). Variations in the thickness of the unit result in variable muting of underlying features such as crater rims, rough bedrock, and fractures. Where observed on the Jezero crater floor, the undifferentiated smooth unit exhibits few small (meters to $\sim10~\mbox{m}$ diameter) craters and fracture networks whose individual polygons are $\sim100\mbox{s}~\mbox{m}$ in diameter (Fig. 8e). It is likely, however, that both the fractures and craters are hosted in the underlying bedrock, and have been thinly mantled by the undifferentiated smooth unit.

Undifferentiated smooth deposits mapped within the study area are generally uniform in tone and texture at map scale, mantle nearly all other units in the map area, exhibit poor retention of craters, and commonly transition gradationally into nearby units. Despite these similarities, these deposits need not be, and are likely not, all time-equivalent, comprised of the same material, or of the same depositional origin. Possible origins include tephra, aeolian deposits, and residual lag accumulations of coarse sand, pebbles, and cobbles due to rock break-down and deflation of the landscape over billions of years. This latter explanation is common in Gale crater, where smooth-surfaced areas on Aeolis Palus identified in HiRISE images were generally observed on the ground to be lags of pebbles weathered out of the underlying conglomeratic bedrock (Stack et al. 2016). Alternatively, occurrences of the undifferentiated smooth unit on the Jezero delta and exposed near the delta’s scarp could be exposures of, or lags left from, eroding friable layers within the deltaic sequence. This interpretation is supported by the appearance of alternating light and dark layers within vertical exposures of the delta sequence. However, distinguishing layers that are inherently dark-toned from the accumulation of dark sand on stair-stepped exposures of layers that are, in actuality, light-toned, is difficult to do at, or even below, map-scale. Thus, distinguishing a deltaic origin for the undifferentiated smooth unit present on the delta from the non-deltaic processes responsible for deposition of this unit elsewhere in the map area is left for future work and/or verification on the surface by the Perseverance rover.

#### Talus (T)

This unit includes accumulations of m-scale boulders resolvable at map scale on dark- to intermediate-toned slopes throughout the map area (Fig. 8f). Boundaries between talus and undifferentiated smooth unit are commonly gradational and approximate, and marked only by a gradual decrease in boulder density. Talus deposits occur predominantly on the crater rim, along the delta front, and on the slopes of isolated buttes and mounds in the map area. These deposits are interpreted to be eroded blocks dislodged and gravitationally displaced from *in-situ* outcrops via physical weathering and aeolian abrasion.

### Bedrock Units: Jezero Crater Floor

The bedrock exposures of the Jezero crater floor described in this section, and those along the inner crater margin described in the next section, presented a particular mapping challenge. These outcrops share textural and tonal similarities that make subdivision difficult, yet they occur over a broad elevation range, areal extent, in potentially diverse depositional settings, exhibit variable relative age relationships to other units in the map area, and are, in some cases, defined by distinct topographic boundaries. In addition, previous studies (e.g., Ehlmann et al. 2008; Goudge et al. 2015, 2017; Horgan et al. 2020) have identified mineralogical distinctions within these bedrock exposures that, while not a criteria for distinguishing units in this map effort, suggest a record of diverse depositional and diagenetic processes. Lumping outcrops of the crater floor and margin into one or two units, as previous studies have done, would have implied a very specific depositional and geologic interpretation that the Mars 2020 Science Team was not prepared to commit to. Thus, to provide the team with a unit nomenclature that would enable discussion and consideration of various depositional and stratigraphic scenarios, the decision was made during reconciliation of map quads to define the units of the Jezero crater floor and inner crater margin primarily by elevation contours that coincided with distinct geographic settings including: the interior margin of the crater, an intermediate elevation interval covering roughly the same elevation range and areal extent as the delta, and those outcrops occurring basinward of the delta. When these elevation-based unit distinctions also coincided with other subtle textural or tonal differences between the units, they are called out in the unit descriptions below.

#### Crater Floor Fractured 1 Unit (Cf-f-1)

The crater floor fractured 1 unit consists of fractured and blocky bedrock that occurs below the −2530 meter elevation contour (Fig. 9a–9c). At map scale, this unit exhibits a mottled tone resulting from a linear mixture of dark and intermediate-toned sand that fills crevices and fractures within bedrock that is primarily light-toned. Exposures appear massive since no stratification can be resolved at map scale. Fractures cross-cutting this unit are, in some places, organized into polygonal networks with individual polygons measuring several meters across (Fig. 9a). Fracturing may derive from a variety of processes including impact (Schultz et al. 1982; Melosh 1989), tectonism (Carr 1974), hydrofracture (Cosgrove 2001), or from contractional stresses associated with thermal cycling or desiccation (Lachenbrauch 1962; Goehring 2013; Oehler et al. 2016).

This unit also forms SW-NE trending ridges, distinct from the polygonal fractures, standing approximately a meter to several meters in high relief that are sometimes aligned with, and sometimes cross-cut by, curvilinear furrows that extend up to 1 kilometer in length (Fig. 9b). Ridge spacing is $\sim50~\mbox{m}$ and the ridge crests vary in length from $\sim200\mbox{--}400~\mbox{m}$. The furrows and ridges do not obviously represent or trace internal stratification, though it is possible that erosion by aeolian abrasion is highlighting subtle differential cementation within stratified bedrock.

The crater floor fractured 1 unit is exposed primarily in two elongate exposures, one near the northern part of the Jezero delta trending NW/SE, and one extending NE/SW near the southern extent of the delta (Figs. 5 and 6). A scarp occurs at the curved contact between this unit and the adjacent crater floor fractured rough unit (Fig. 9c). The crater floor fractured 1 unit appears to underlie the adjacent crater floor fractured rough unit in the immediate vicinity of the contact, although the exposed surface of the crater floor fractured 1 unit exhibits topographic relief up to 40 m, but more commonly between 10–20 m, above the adjacent crater floor fractured rough unit.

Goudge et al. (2015) included the crater floor fractured 1 unit within their LTF unit and interpreted it to be stratigraphically equivalent to carbonate and olivine-bearing light-toned fractured rocks that occur around the inner rim of Jezero crater (MT unit) and outside the crater rim. Numerous interpretations have been proposed for this regionally-extensive rock unit including: lava flows (Tornabene et al. 2008; Ody et al. 2013), magmatic intrusions (Hoefen et al. 2003), impact condensates (Palumbo and Head 2018; Rogers et al. 2018), tephra deposits (Bramble et al. 2017; Kremer et al. 2019; Mandon et al. 2020), aeolian, and fluvial deposits (Rogers et al. 2018). Given the context of this unit as a fill within the Jezero crater basin, and lacking an obvious extrusive volcanic source (vent or edifice) within or near the crater, an origin as volcanic ash or airfall, aeolian, or fluvio-lacustrine sediments seems most plausible.

#### Crater Floor Fractured 2 Unit (Cf-f-2)

The crater floor fractured 2 unit consists of fractured, blocky bedrock that crops out between the $-2530~\mbox{m}$ and $-2440~\mbox{m}$ elevation contours in the western portion of the Jezero crater floor (Fig. 9d). Fractures that cut rocks of this unit are rectilinear to subpolygonal with individual polygons measuring several meters across. Sets of large ($\sim10^{2}~\mbox{m}$), arcuate fractures are also observed. This unit appears massive, i.e., no indications of internal stratification. The crater floor fracture 2 unit is similar to the crater floor fractured 1 unit in both tone and texture, but it is subtly distinguished by a rougher, pock-marked surface texture resulting from the presence of small m-scale bumps and ridges (Fig. 9d). This unit also exhibits some textural and tonal similarities to the crater floor fractured rough (Cf-fr) unit described below, but the crater floor fractured 2 unit retains fewer craters and lacks the distinctive resistant curved margins of the crater floor fractured rough unit. The contacts between the crater floor fractured 2 unit, the lower elevation crater floor fractured 1 unit, and the higher elevation margin fractured unit are all gradational. The crater floor fractured 2 unit is also in contact with the Jezero delta, with $\sim40~\mbox{m}$ of relief on the contact between the crater floor fractured 2 unit and the layered deposits of the western delta.

This unit has the same range of published interpretations as the crater floor fractured 1 unit, since previous studies have not distinguished these two units. As with the crater floor fractured 1 unit, Goudge et al. (2015) interpreted the crater floor fractured 2 unit to be stratigraphically equivalent to carbonate and olivine-bearing light-toned fractured rocks that occur around the inner rim of Jezero crater and that drape and extend outside the crater rim as part of a regional olivine- and carbonate-bearing unit. As such, origins as volcanic ash or airfall, aeolian, or fluvio-lacustrine sediments seem to be most plausible. Given the direct contact between the crater floor fractured 2 unit and the Jezero delta and their equivalent elevation ranges, lacustrine or deltaic interpretations may be particularly compelling for the crater floor fractured 2 unit compared to crater floor fractured 1, although the gradational transition between these two units and their textural similarities suggests similar depositional origins.

#### Crater Floor Fractured Rough Unit (Cf-fr)

The crater floor fractured rough unit is light- to medium-toned, rough on the meter-scale, boulder-producing, and crater-retaining (Fig. 9e and 9f). By comparison to other bedrock units within Jezero crater, it is the most crater-retaining unit (Goudge et al. 2015). The craters are all interpreted to have formed by exogenic impact processes and range from craters $<10~\mbox{m}$ in diameter to craters ranging in size from 10–100 meters in diameter. This unit contains fractures at two distinct length scales: small fractures forming polygons up to a few meters across (Fig. 9e) and large fractures with lengths up to several hundreds of meters (Fig. 9f). The polygonal fractures are linear to arcuate in form and occur in two distinct topographic forms: (a) in negative relief as shallow indentations in the substrate, or (b) in positive relief as raised ridges with central indentations (i.e., a double ridge) (Fig. 9f). Fractures commonly transition between relief types along the length of the fracture. This unit is relatively planar in expression with local relief of only a few meters. In comparison, the crater floor fractured 1 and 2 units exhibit undulating and variable relief on the scale of tens of meters.

The crater floor fractured rough unit comprises much of the Jezero crater floor and the eastern portion of the map area. Large expanses of this unit appear to be overlain by and exposed between deposits of the undifferentiated smooth unit, and the contact between these two units often appears gradational. Where this unit is observed to be covered by the undifferentiated smooth unit, fewer fractures, craters, and rough textures are observed. The contact between the crater floor fractured rough unit and the underlying crater floor fractured 1 unit is marked by a curving scarp, sometimes expressed as a series of resistant ridges, that highlights a topographic distinction between these two units.

This unit is interpreted as lithified bedrock, in contrast to the undifferentiated smooth unit that overlies it, which is interpreted as an unconsolidated surface mantle. Goudge et al. (2015) and Schon et al. (2012) interpreted the rocks of the crater floor fractured rough unit to be a basaltic lava flow that resurfaced the Jezero crater floor. This interpretation was based primarily on visual similarities, e.g., dark tone and high crater retention, to their perspective of what lava flows look like elsewhere on Mars. However, observations via the Curiosity rover, in concert with HiRISE images of terrain in Gale crater, have shown that well-cemented sandstones (e.g., Edgett and Malin 2014) and even well-cemented mudstones (Calef et al. 2019) can retain many sub-kilometer-scale impact craters per surface unit area. As noted by Edgett (2018), some crater-retentive sedimentary rock units could otherwise be confused as lava plains. Thus, fluvial and aeolian sedimentary origins are also plausible interpretations for the crater floor fractured rough unit in Jezero crater. The dark tone that was associated by previous researchers with this unit is disassociated from the bedrock and is instead the result of the partial superposition by the undifferentiated smooth unit. Where surficial cover is thinner, the crater floor fractured rough exposures are lighter in tone.

### Bedrock Units: Jezero Crater Inner Margin

#### Margin Fractured Unit (M-f)

The margin fractured unit encompasses exposures of light-toned fractured bedrock along the inner margin of Jezero crater between the elevation contours of $-2440~\mbox{m}$ and $-2190~\mbox{m}$ south of Neretva Vallis and $-2440~\mbox{m}$ and $-2240~\mbox{m}$ north of Neretva Vallis (Fig. 10). Local brightness variations within this unit correlate with apparent m-scale surface roughness and textures such as polygonal patterns of fractures, ridges $\sim10~\mbox{m}$ in length, and exposures of erosionally resistant blocks between 1–5 m in diameter. Two dominant surface expressions of this unit include blocky, ridge-forming outcrops (Fig. 10a) and low relief, less blocky textures (Fig. 10b). Fractures cross-cut both expressions and are observed to continue uninterrupted from one expression to the other. The small ridges and cliffs within the blocky, ridged outcrops trend northeast/southwest and are composed of dislodged and displaced polygonal bedrock blocks. Locally, low-relief, less blocky outcrops often occur topographically below the blocky, ridged exposures, but both expressions occur over nearly the full elevation range of the unit without the obvious appearance of being interbedded or layered. Since these observed surface expressions could not be consistently mapped as subunits representing true rock volumes, the decision was made not to subdivide the margin fractured unit. Generally, this unit appears massive, i.e., stratification is not observed. The margin fractured unit retains some craters, though not as extensively as the crater floor fractured rough unit. Though fractured into blocks that are 1–5 m in diameter, this unit does not exhibit the 100 m-scale arcuate or northeast/southwest trending fractures observed in the crater floor fractured 1 and 2 units.

The margin fractured unit is in contact with, and appears to locally underlie, the delta blocky unit (Fig. 10c). The contact between the margin fractured unit and the crater floor fractured 2 unit is gradational; morphologically, these two units are very similar. The margin fractured unit was interpreted by Ehlmann et al. (2008) and Goudge et al. (2015) as spatially continuous with an extensive carbonate- and olivine-bearing unit that superposes the Jezero crater rim and extends north, west, and southwest beyond the crater. Numerous interpretations have been proposed for this regionally-extensive deposit including: lava flows (Tornabene et al. 2008; Ody et al. 2013), magmatic intrusions (Hoefen et al. 2003), impact condensates (Palumbo and Head 2018; Rogers et al. 2018), tephra deposits (Bramble et al. 2017; Kremer et al. 2019; Mandon et al. 2020), aeolian, and fluvial deposits (Rogers et al. 2018). Alternatively, Horgan et al. (2020) proposed that the margin fractured unit may be an authigenic carbonate-bearing deposit formed in a near-shore lacustrine environment. This study stops short of identifying a preferred interpretation for this unit, as the depositional interpretation is largely context-dependent as discussed in greater detail in the sections that follow.

### Bedrock Units: Jezero Crater Delta

#### Delta Blocky Unit (D-bl)

The delta blocky unit is an intermediate-toned deposit characterized by a variegated texture due to the presence of blocks of variable tone and size resolvable at map scale (Fig. 11a). The delta blocky unit can form steep-sided boulder-shedding mesas, mounds, and terraces, and positive relief elongate ridges 100–300 meters in width and a few tens of meters high on the delta’s top surface that alternate with troughs in which large and small aeolian bedforms and undifferentiated smooth unit accumulate. The margins of this unit are defined by small scarps, but where this unit is in contact with the Us, the transition is diffuse. Although it is difficult to determine from orbiter image data whether this unit is indurated, it is coherent enough to form and maintain ridges and scarps that are organized into several discernible overlapping triangular deposits, interpreted as depositional lobes whose proximal apex is the avulsion node (Stack et al. 2020). This unit is interpreted as inverted coarse-grained fluvial channel deposits, consistent with the past interpretations by Fassett and Head (2005), Schon et al. (2012), and Goudge et al. (2018). This unit appears to overlie the delta truncated curvilinear layered unit and locally the delta thick and thinly layered units.

#### Delta Thinly Layered Unit (D-tnl)

The delta thinly layered unit consists of a stratified sequence of alternating light and dark bands, each $<1~\mbox{m}$ in apparent thickness, that appear planar and approximately horizontal and are continuously traceable over length scales of up to several hundreds of meters (Fig. 11b). Locally contorted and folded light-toned layers are observed (Fig. 11b), as well as layers that exhibit an irregular, scalloped and corrugated edge resulting in a “lacy” texture where dark-toned deposits occur in round to sub-rounded patches on/within light-toned bedding planes that are exposed in plan view. Polygonal fractures are sometimes observed within the light-toned layers. The dark interbeds between the light-toned layers could be actual dark-toned rock layers, or could be dark sand or mantling deposits that accumulated on stair-stepped light-toned ledges.

The delta thinly layered unit is observed primarily along the base of the scarp that defines the southeastern edge of the western delta, and appears to be consistently stratigraphically and topographically below the delta blocky unit. The relationship between this unit and the delta truncated curvilinear layered unit, which sometimes occur at equivalent elevations, is less clear. The delta thinly layered unit is distinguished from the delta thick layered unit, described below, by the increased proportion and prominence of dark, smooth interlayers, as well as the apparent thickness of the layers. The delta thinly layered unit also occurs in remnant mounds and mesas east of the main western delta deposit that are interpreted here and by Schon et al. (2012) and Goudge et al. (2015) to be remnants of a formerly more extensive delta or lacustrine deposit (Fig. 11c). Schon et al. (2012) interpreted this unit as being part of the delta plain sequence of alluvial sediments and floodplain deposits. In contrast, Goudge et al. (2017) interpreted this unit to be fine-grained bottomset beds deposited in a prodelta setting. Tice et al. (2020) interpreted this unit as a more distal facies representing hemipelagic deposition in the Jezero basin contemporaneous with delta deposition.

#### Delta Thickly Layered Unit (D-tkl)

The delta thickly layered unit is composed of light-toned, rough-textured, erosionally resistant layers (Fig. 11d). Individual layers measure up to several meters thick, in contrast to the layers of the delta thin layered unit which are typically $<1~\mbox{m}$. Light-toned layers within the delta thick layered unit are traceable for 100s of meters without evidence of truncation or pinch outs, and appear approximately horizontal. The delta thickly layered unit is exposed on cliff faces and caps along the northeastern margin of the western delta deposit, and along the base of several remnant mounds east of the western delta deposit. The delta thickly layered unit appears to be locally stratigraphically below the delta blocky unit. The delta thickly layered unit occurs at a higher elevation than the delta thinly layered unit, but these two units are not observed to be in direct contact with each other.

This unit is interpreted to be likely coarser-grained and deposited in a more proximal setting than the underlying delta thinly layered unit given its relatively greater resistance to erosion and rougher, blocky-weathering texture. Schon et al. (2012) interpreted this unit to be alluvial or flood plain deposits from a delta plain setting while Goudge et al. (2017) interpreted the lower layers of this unit to be bottomset beds deposited in a prodelta setting and its upper layers as shallowly dipping delta front foresets. Tice et al. (2020) interpreted the resistant light-toned beds within this unit as channel lobes formed at the toe of the delta slope.

#### Delta Truncated Curvilinear Layered Unit (D-tcl)

The delta truncated curvilinear layered unit consists of decimeter-scale sets of alternating light- and dark-toned strata that truncate against one another over length-scales of tens of meters (Fig. 11e). These sets are bounded by laterally continuous layers that truncate against one another over scales of hundreds of meters. The delta truncated curvilinear layered unit is exposed primarily on the top surface of the delta in local topographic lows between exposures of the delta blocky unit. The delta truncated curvilinear layered unit exhibits minimal vertical exposure and is typically exposed in horizontal plan view outcrops. This unit locally appears to be topographically and stratigraphically below the delta blocky deposits, but is elevation-equivalent to the delta thinly layered unit in the southern portion of the delta and to the delta thickly layered unit in the northeastern portion of the delta.

The delta truncated curvilinear layered unit was interpreted as laterally accreting point bars deposited by meandering fluvial channels in a delta plain environment (Ehlmann et al. 2008; Schon et al. 2012; Goudge et al. 2017, 2018). Tice et al. (2020) interpreted this unit to have formed in a proximal to medial subaqueous delta slope setting, with truncated curvilinear sets representing subaqueous channel-levee complexes, terminal mouth bars, and unconfined flow deposits.

#### Delta Layered Rough Unit (D-lr)

The delta layered rough unit is characterized by light-toned, parallel, m-thick layers exhibiting a rough surface texture (Fig. 11f). This unit is distinguished from layered deposits elsewhere in the map area by their lighter tone and mottled surface texture. The delta layered rough unit crops out exclusively along slopes and cliffs in the northeastern fan deposit adjacent to the western delta. Individual layers are traceable for $\sim100$ meters, and no truncations are visible.

This unit was interpreted by Fassett and Head (2005) and Goudge et al. (2015) to have been deposited by a different fan system sourced from Sava Vallis incised into the northern rim of Jezero crater. This study observes no transport indicators that would distinguish a northern versus western source for this deposit.

### Bedrock Units: Jezero Crater Rim and Beyond

#### Crater Rim Blocky Unit (Cr-bl)

The crater rim blocky unit is intermediate-toned and forms erosionally resistant high-standing ridges that erode to form boulders (Fig. 12a). Exposures of this unit exhibit a m-scale rubbly texture at the map scale as a result of these boulder accumulations, and appear massive with no evidence for internal layering at map scale. The crater rim blocky unit is discontinuous and exposed in patches; these cover areas ranging from a few 10s of m across to more areally extensive regions of 100s of m across. Ridges comprised of this unit vary from 10s to 100s of meters in length, and form the high-standing portions of the Jezero crater rim. The majority of the exposed crater rim is composed of this unit, and it is not observed elsewhere except for the crater rim. This unit is interpreted to represent pre-impact bedrock that uplifted during the Jezero impact to form the crater rim.

#### Crater Rim Breccia Unit (Cr-br)

The crater rim breccia unit includes occurrences of brecciated and disrupted light- and intermediate-toned bedrock exposed on the Nili Planum-facing slope of the Jezero crater rim both north and south of Neretva Vallis (Fig. 12b). Individual blocks measure 10 to $>100~\mbox{m}$ in diameter. Hints of faint stratification are observed in exposures of crater rim breccia, although deformation and brecciation is interpreted to have obscured or destroyed much of the bedrock’s primary fabric. The crater rim breccia unit occurs at equivalent elevations as the crater rim layered unit along the outwards slope of the crater rim, and crops out within the elevation range of crater rim blocky unit exposures mapped on the inward Jezero-facing slope of the crater rim.

The crater rim breccia unit is interpreted as impact breccia, though it is uncertain whether this breccia was formed during the Jezero impact event, or is an occurrence of the syn-Isidis megabreccia (Mustard et al. 2009; Bramble et al. 2017; Scheller and Ehlmann 2020) within the pre-Jezero basement sequence that was uplifted during the Jezero impact.

#### Crater Rim Layered Unit (Cr-l)

The crater rim layered unit displays a light tone and exhibits meter to sub-meter thick layers when observed in cross-section. The unit also contains fractured-bounded polygons that range from meters to tens of meters across, though these fractures are less prominent than in the fractured units infilling Jezero or those observed in the Nili Planum fractured unit. Layered exposures show occasional faulting and folding (Fig. 12c). This unit is often partially mantled by the undifferentiated smooth unit and appears to locally underlie the crater rim blocky unit. The crater rim layered unit occurs predominantly within and along the outside edge of the Jezero crater rim, although it also appears to crop out on the rim itself in erosional windows below the crater rim blocky unit.

This unit is interpreted to be part of the bedrock sequence that predates the formation of Jezero crater, uplifted by the Jezero impact and further exposed by subsequent erosion. The unit’s stratification points to a likely sedimentary or explosive volcanic origin.

#### Crater Rim Rough Unit (Cr-r)

The crater rim rough unit exhibits a light tone, high crater retention, and characteristic m-scale rough texture (Fig. 12d). This unit’s variegated tone is caused by dark sand irregularly infilling small pits; dark-toned sediment lags also serve to enhance the surface’s rough texture. Coarse, meter-scale stratification is observed along the edges of crater rim rough unit where it crops out. Morphologically, this unit is very similar to the crater floor fractured rough unit inside Jezero and shares a similar erosional expression defined at its edges by curved scarps. It also appears similar in morphology to the so-called mafic capping unit (Bramble et al. 2017) identified in the Nili Planum (informally northeast Syrtis) region (Sun and Stack 2019). This unit occurs in one specific location on the crater rim within the mapped area, where it overlies the crater rim blocky unit, but its relationship to either the Nili Planum capping unit or the Jezero crater floor fractured rough unit is uncertain. As such, there are few clues to this unit’s origin, though its occurrence draping the Jezero crater rim could suggest deposition by sedimentary or explosive volcanic processes.

#### Neretva Vallis Layered Unit (NV-l)

The Neretva Vallis layered unit is composed of light- to intermediate-toned layered outcrops exhibiting m-scale fracture-bounded polygons (Fig. 13a–13c), often with a better-defined reticulate pattern and narrower crack widths than other fractured units observed elsewhere throughout the map area, particularly those observed on the Jezero crater floor. This unit occurs as outcrops $10^{2}\mbox{--}10^{3}~\mbox{m}^{2}$ in area exposed intermittently within the Neretva Vallis walls and floor, and is not observed in Nili Planum or within Jezero crater. Outcrops exposed along the walls of Neretva Vallis could have been deposited within the channel by fluvial processes, or could be exposed bedrock into which the valley incised. Exposures of the Neretva Vallis layered unit observed on the valley floor are distinct enough from the surrounding Nili Planum fractured unit, particularly given the presence of clear layering, that an interpretation as a likely lithified fluvial sedimentary deposit formed during Neretva Vallis incision is favored.

#### Nili Planum Fractured Unit (NP-f)

The Nili Planum fractured unit consists of light-toned fractured outcrop west of the Jezero crater rim, both north and south of Neretva Vallis (Fig. 13d and 13e). This unit is characterized by a m-scale rough surface texture and sub-rectilinear/fracture polygons up to $\sim20~\mbox{m}$ across. This unit commonly preserves impact craters and, in places, has eroded to form boulders. Stratification is not obvious at map scale, and a blocky, massive expression is most common (Fig. 13e), although low-relief exposures lacking the blocky expression are also observed (Fig. 13d). Morphologically, this unit appears very similar to the crater floor fractured 1 and 2 units and the margin fractured unit within Jezero crater.

This unit is commonly found on Nili Planum outside of Jezero crater north and south of Neretva Vallis. Similarities between the Nili Planum fractured unit and the olivine and carbonate-bearing light-toned fractured deposits observed beyond this study’s map area elsewhere in Nili Planum (Ehlmann and Mustard 2012; Goudge et al. 2015; Bramble et al. 2017; Mandon et al. 2020) and that are observed to drape the Jezero crater rim (Goudge et al. 2015) suggest that the Nili Planum fractured unit is younger than the bedrock units that make up the Jezero crater rim. If the Nili Planum fractured unit is part of the olivine and carbonate-bearing unit exposed throughout this region as interpreted by Goudge et al. (2015), then the origins proposed for this regionally extensive unit would be possible explanations for the Nili Planum fractured unit as well, including: lava flows (Tornabene et al. 2008; Ody et al. 2013), magmatic intrusions (Hoefen et al. 2003), impact condensates (Palumbo and Head 2018; Rogers et al. 2018), tephra deposits (Bramble et al. 2017; Kremer et al. 2019; Mandon et al. 2020), aeolian, and fluvial deposits (Rogers et al. 2018).

## Correlation of Map Units

### Jezero Crater Rim and Beyond

The rock units exposed on the Jezero crater rim, specifically the crater rim blocky unit, the crater rim breccia unit, and the crater rim layered unit, are interpreted to be the oldest units within the mapped area. Given their exposures within the Jezero rim, the crater rim blocky deposit and crater rim layered unit likely pre-date the impact event that formed Jezero crater. The crater rim breccia unit may also predate the Jezero impact, although a syn-Jezero formation age cannot be conclusively ruled out at this time. The Nili Planum fractured unit and the crater rim rough unit appear to onlap and drape the crater rim, respectively, so both units are interpreted to be younger than the crater rim blocky, crater rim breccia, and crater rim layered units. Neretva Vallis incises the crater rim units as well as the Nili Planum fractured unit, so the Neretva Vallis layered unit is interpreted to be the youngest bedrock unit outside the crater. The Neretva Vallis layered unit is interpreted to be generally coeval with deposition of the Jezero delta, but the precise timing of the Neretva Vallis layered unit deposition relative to specific units of the Jezero delta units is not well constrained.

### Jezero Crater Interior

Based on superposition and cross-cutting relationships, the oldest exposed unit within Jezero crater is the crater floor fractured 1 unit, followed by the crater floor fractured 2 unit. The crater floor fractured rough unit, as well as the units that make up the delta, locally appear to overlie the crater floor fractured 1 and 2 and the margin fractured units, although alternate age relationships and correlations with units outside Jezero crater are explored in the four correlation scenarios described below. These scenarios are not the only correlations possible for the map area, but they represent endmember models that convey the primary relative age relationships between the major units, while also highlighting which interpreted age relationships have the greatest uncertainty at the present time.

#### Scenario 1

**Fig. 14:**
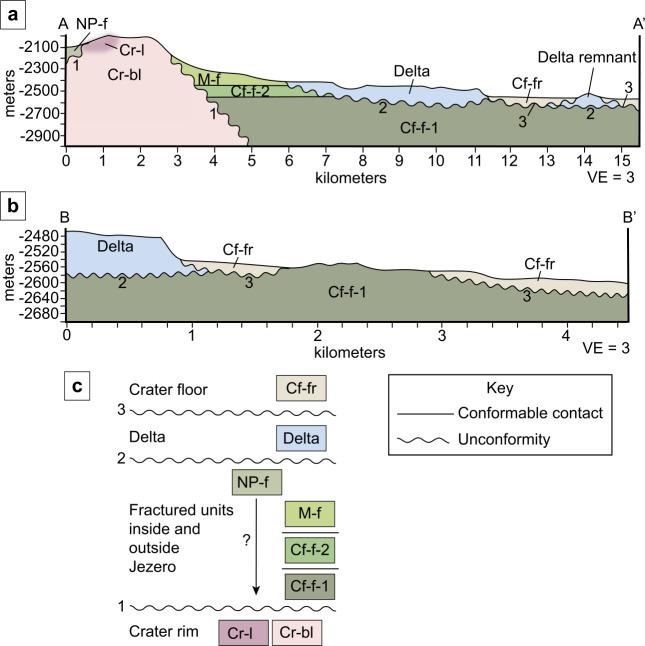
(**a**) Cross-section A to A’ showing interpreted unit correlation for Scenario 1. Numbers correspond to unconformities identified in (**c**). (**b**) Cross-section B to B’ showing interpreted unit correlation for Scenario 1. Numbers correspond to unconformities identified in (**c**). (**c**) Schematic unit correlation representing unit relationships shown in (**a**) and (**b**). For simplicity, the western Jezero delta, the fan deposit northeast of the western delta, and remnants mounds are shown here as a single “Delta” group

In Scenario 1 (Fig. 14), the crater floor fractured 1 and 2 units and the margin fractured unit within Jezero are shown as a conformable sequence deposited in time order according to their respective elevations. These three fractured units within Jezero are shown as possibly coeval and correlative with the Nili Planum fractured unit outside of Jezero, all of which are preceded in age by the units comprising the crater rim. The units of the Jezero delta, considered here to be a single depositional sequence for relative simplicity, would have been deposited unconformably on the crater floor fractured 1 and 2 units and the margin fractured unit, extending to the east at least as far as the easternmost preserved remnant mound. Following the draining and drying of the Jezero crater lake and erosion of the delta to its present-day extent, deposition of the crater floor fractured rough unit would have occurred, embaying the delta and its remnants as well as the eroded, exposed outcrop of the underlying crater floor fractured units. Deposition and accumulation of the undifferentiated smooth unit and more recent aeolian bedforms throughout the mapped area would complete the scenario. This unit correlation recognizes three major unconformities within the bedrock sequence mapped in and around Jezero crater (Fig. 14c): one between the bedrock units that comprise the Jezero crater rim and the overlying Nili Planum fractured unit and the oldest units infilling Jezero (crater floor fractured 1 and 2 units and the margin fractured unit), a second between the delta and its remnants and the underlying margin fractured and crater fractured 1 and 2 units, and a third between the delta and its remnants and the crater floor fractured rough unit.

#### Scenario 2

**Fig. 15:**
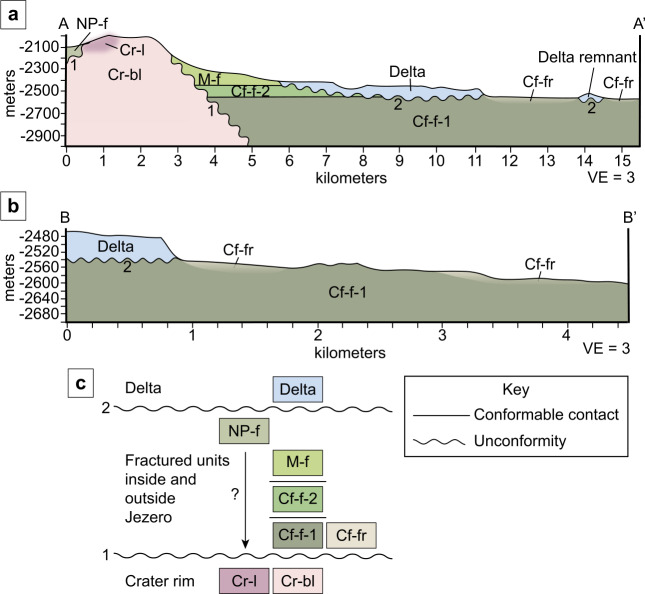
(**a**) Cross-section A to A’ showing interpreted unit correlation for Scenario 2. Numbers correspond to unconformities identified in (**c**). (**b**) Cross-section B to B’ showing interpreted unit correlation for Scenario 1. Numbers correspond to unconformities identified in (**c**). (**c**) Schematic unit correlation representing unit relationships shown in (**a**) and (**b**). For simplicity, the western Jezero delta, the fan deposit northeast of the western delta, and remnants mounds are shown here as a single “Delta” group

Scenario 2 (Fig. 15) is similar to Scenario 1 in that the crater floor fractured 1 and 2 units and the margin fractured unit within Jezero are shown as a conformable sequence that is possibly correlative and coeval with the Nili Planum fractured unit outside of Jezero. As in Scenario 1, the Jezero delta and its remnants are unconformably overlain on the crater floor and margin fractured units. However, unlike Scenario 1, Scenario 2 includes the crater floor fractured rough unit within the same depositional sequence as the other intra-Jezero fractured units in recognition of the textural and tonal similarities between the crater floor fractured rough unit and the other fractured units within Jezero, and the exposure of the crater floor fractured rough unit within the same elevation range as the crater floor fractured 1 unit. Following the deposition and some erosion of the fractured units both inside and outside of Jezero, Scenario 2 shows the deposition of the Jezero delta extending at least to the easternmost remant.

This unit correlation implies an unconformity between the bedrock units that comprise the Jezero crater rim and the fractured units inside and outside the crater (Fig. 15c). A second unconformity would occur within the Jezero infilling sequence between the delta units and the sequence of fractured units within Jezero. In this scenario, the delta units are the youngest bedrock within Jezero and are among the youngest units in the mapping area.

#### Scenario 3

**Fig. 16:**
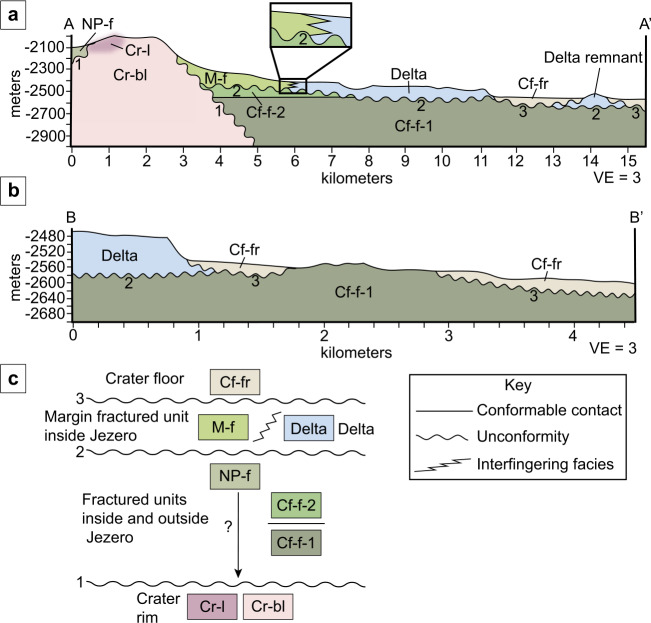
(**a**) Cross-section A to A’ showing interpreted unit correlation for Scenario 3. Numbers correspond to unconformities identified in (**c**). (**b**) Cross-section B to B’ showing interpreted unit correlation for Scenario 1. Numbers correspond to unconformities identified in (**c**). (**c**) Schematic unit correlation representing unit relationships shown in (**a**) and (**b**). For simplicity, the western Jezero delta, the fan deposit northeast of the western delta, and remnants mounds are shown here as a single “Delta” group

Scenario 3 (Fig. 16) recognizes the potential of an interfingering relationship between the delta and the adjacent, elevation-equivalent margin fractured unit. Unlike Scenario 1, Scenario 3 shows the margin fractured unit inside the crater as distinct from and unconformable with the other fractured units within Jezero. In this scenario, the margin fractured unit and the Jezero delta units would represent interfingered shallow lacustrine and deltaic facies, respectively. Deposition of the underlying crater floor fractured 1 and 2 units could have occurred in an ancient Jezero lake, or deposition of these units along with the potentially correlative Nili Planum fractured unit could have entirely pre-dated the presence of a lake within Jezero. Following the draining and drying of the Jezero crater lake, Scenario 3 shows the deposition of the crater floor fractured rough unit embaying the eroded delta and margin and crater floor fractured 1 and 2 units.

This scenario recognizes an unconformity between the bedrock units of the crater rim and the oldest fractured units deposited inside and outside Jezero (Fig. 16c). A second unconformity would exist between the crater floor fractured 1 and 2 units and the overlying interfingered margin fractured unit and delta sequence. A third significant unconformity within the Jezero infilling sequence would be between the crater floor fractured rough unit and the units it embays: the interfingered sequence of margin fractured unit and the delta and the crater floor fractured 1 and 2 units.

#### Scenario 4

**Fig. 17:**
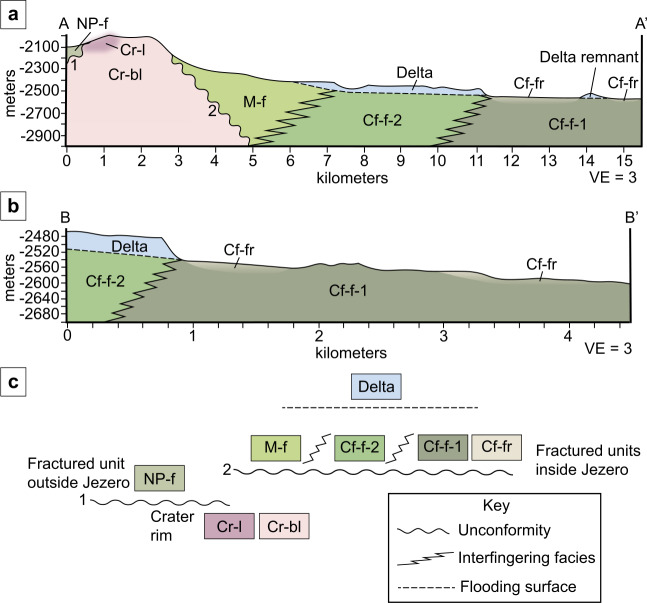
(**a**) Cross-section A to A’ showing interpreted unit correlation for Scenario 4. Numbers correspond to unconformities identified in (**c**). (**b**) Cross-section B to B’ showing interpreted unit correlation for Scenario 1. Numbers correspond to unconformities identified in (**c**). (**c**) Schematic unit correlation representing unit relationships shown in (**a**) and (**b**). For simplicity, the western Jezero delta, the fan deposit northeast of the western delta, and remnants mounds are shown here as a single “Delta” group

Scenario 4 (Fig. 17) shows the delta, margin, and crater floor fractured units as part of the same depositional sequence with no major unconformities within it. As in Scenario 2, the crater floor fractured rough unit is considered part of the crater floor fractured 1 unit, but Scenario 4 shows the margin fractured and crater floor fractured 1 and 2 units as interfingering, time equivalent facies, rather than as lithostratigraphic units deposited in series as in Scenarios 1–3.

In Scenario 4, deposition of the Nili Planum fractured unit would have occurred after the formation of Jezero; this unit may or may not have also filled Jezero. At some time later, the interfingered fractured units would have been deposited within the Jezero lake representing time-equivalent proximal to distal lacustrine facies. The fractured units exposed in the crater floor today could have been interfingered with older delta deposits further out into the basin, now eroded away or buried below the present-day crater floor, or they may have pre-dated delta deposition altogether. A sudden rise in lake level would have resulted in back-stepping of the depositional system, with deposition of the western Jezero delta observed today proximal to the source near the crater rim.

This scenario (Fig. 17c) recognizes significant unconformities between the crater rim bedrock and the Nili Planum fractured unit, and between the crater rim bedrock and the Jezero infilling units. Some erosion could have occurred at the flooding surface shown between the delta and underlying fractured units, but the relative time implied by this surface is significantly less than that implied by the major unconformities in this and other correlation scenarios.

### Jezero Delta

**Fig. 18:**
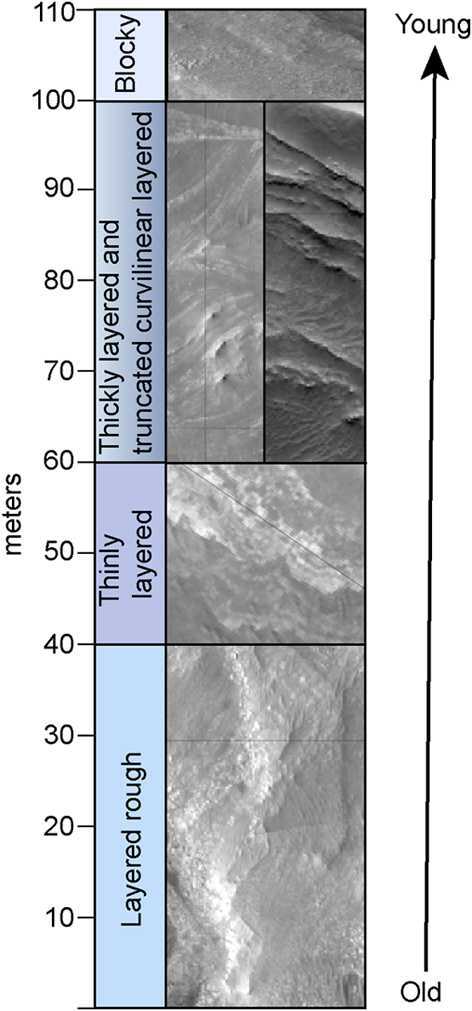
Relative stratigraphic order and approximate thickness of units mapped within the Jezero delta

Several consistent relative age relationships are observed between the units that compose the Jezero delta (Fig. 18). The delta blocky unit is observed to overlie the delta layered rough unit, delta truncated curvilinear layered unit, and the delta thickly and thinly layered units, suggesting that it is the youngest of the delta bedrock units. The relative age relationship between the truncated curvilinear layered unit and the thickly layered unit is less clear. Both the thickly layered unit and the truncated curvilinear layered unit occur locally at equivalent elevations, so they each may represent time equivalent facies deposited in different depositional settings. While the thickly layered unit and the thinly layered unit are not in direct contact with each other, the thinly layered unit is consistently observed below exposures of the truncated curvilinear unit which suggests that the thinly layered unit is older than both the truncated curvilinear layered unit and the thickly layered unit.

There is some uncertainty in the age of the delta layered rough unit, which occurs exclusively to the northeast of the western delta. The delta layered rough unit crops out at the lowest elevation of all the delta deposits which, if used as a proxy for age, could represent the oldest deposit within the delta. However, this unit only has a clear contact with the overlying delta blocky unit, so its relationship to the delta thinly layered, thickly layered, and truncated curvilinear layered units remains uncertain.

## Discussion

### Comparisons with Previous Mapping Efforts

The 1:5000 scale Mars 2020 Science Team photogeologic map represents the most detailed and comprehensive mapping effort of this area to-date. Goudge et al. (2015), the only other published map that covers the same area mapped in this study, was mapped at 1:30,000 and using a CTX image mosaic. It is not surprising, then, that this study’s map resolves noticeably more detail in the mapped contacts than the map in Goudge et al. (2015). Despite the differences in scale, the locations of the main bedrock units are generally consistent. Goudge et al. (2015) and this study recognized that much of the crater rim and wall is a single unit (crater rim blocky unit), though this study resolves the crater rim layered unit from those that appear massive and blocky (crater rim blocky unit and breccia unit). Goudge et al. (2015) and this study also identified an extensive crater floor unit; Goudge et al.’s (2015) “Volcanic floor unit” covers approximately the same extent as this study’s crater floor fractured rough unit. Goudge et al. (2015) and this study also both made a distinction between the fractured units within Jezero crater, separating the lower-elevation unit exposed in the curved inliers within the crater floor (Goudge et al.’s (2015) “light-toned floor unit,” this study’s crater floor fractured 1 and 2 units) and the margin unit (Goudge et al.’s (2015) “mottled terrain,” this study’s margin fractured unit), with this study distinguishing an additional unit, crater floor fractured 2 unit, based on both elevation and subtle textural differences. The spatial extent of this study’s margin fractured unit within the crater generally matches Goudge et al.’s (2015) “mottled terrain,” although this study splits the fractured units outside the crater from those within the crater, enabling consideration of alternative unit correlations than that presented in Goudge et al. (2015).

The most obvious difference between this study’s map and that of Goudge et al. (2015) is the finer level of detail employed in mapping the spatial extent and boundaries of surficial deposits including the large and small aeolian bedforms and the undifferentiated smooth unit. The spatial scale employed here is necessary for strategic planning of the Perseverance mission in Jezero crater. Although the Goudge et al. (2015) map included a “surficial debris cover” unit within Jezero crater, this study recognizes extensive smooth deposits (mapped as undifferentiated smooth unit) that occur on the crater floor, the delta, and the crater rim. This study’s map shows fields of aeolian bedforms that cover major expanses of the inner margin of Jezero crater and low-relief units exposed on the crater floor, commonly obscuring underlying bedrock and possible unit contacts nearly completely. Talus accumulations occur predominantly along the steep front of the delta, on the slopes of the remnant mounds, and in isolated occurrences on the crater rim where boulders shed from the crater rim blocky unit.

This study also maps the Jezero delta in increased detail compared to previous studies. Goudge et al. (2015) maps the western Jezero delta as a single unit, recognizing only the large impact crater (Belva crater) and the northeastern deposit as additional distinct units. This study does not distinguish a specific unit for Belva crater as Goudge et al. (2015) did as no impact deposits such as ejecta or breccia were observed, but does map it as exposing part of the delta truncated curvilinear layered unit that is observed elsewhere within the delta. Like Goudge et al. (2015), this study recognizes the fan deposit to the northeast of the western delta (delta layered rough unit) as distinct from the units present within the rest of the western delta. Goudge et al. (2015) interpreted this deposit to originate from Sava Vallis, but this study finds no obvious indication within the map area and at map scale for a north-to-south versus an east-to-west sediment transport direction.

This study’s distinction of units within the western delta is similar to the map of Goudge et al. (2018), which recognizes three units within the western delta: point bar strata, inverted channel bodies, and the inlet valley. Goudge et al.’s (2018) “point bar strata” unit generally coincides with this study’s delta truncated curvilinear layered unit and “inverted channel bodies” generally maps to this study’s delta blocky unit. Schon et al.’s (2012) “channel deposits” also maps closely to this study’s delta blocky unit. Ehlmann et al. (2008), Schon et al. (2012), and Goudge et al. (2015, 2017, and 2018) all recognized the presence of stratified material within the Jezero delta, although none show their full extent on published maps. This study’s map also recognizes the presence of stratified rock, as well as deposits most similar to the delta blocky unit, within the remnant mounds. The relative age relationship of delta units resulting from this study is generally consistent with that proposed by Ehlmann et al. (2008), who observed a sequence of layered deposits overlain by the truncated curvilinear layered unit (referred to as the “point bar facies”), and capped by the delta blocky unit.

### Unit Correlations

Of the four correlations considered for the mapped study area, Scenario 1 (Fig. 14), which recognizes significant unconformities between the delta and the margin/crater floor fractured 1 and 2 units and between the delta and the crater floor fractured rough unit, is most consistent with the previous interpretations of Ehlmann et al. (2008) and Goudge et al. (2015). Although this study does not find strong evidence to reject this scenario, the distribution of units mapped in this study and the additional detailed unit characterization presented here encourages consideration of the three alternative interpretations.

Previous interpretations of a significant unconformity between the crater floor fractured rough unit and the crater floor fractured 1 and 2 units, were based, in part, on differences in the tone (dark versus light) and the sharp topographic boundary between the crater floor fractured rough unit and the adjacent crater floor fractured 1 and 2 units. This study’s map recognizes this distinct topographic break, but also the striking textural and tonal similarities between the crater floor fractured 1 unit and the crater floor fractured rough unit (Figs. 9 and 10). Additionally, the crater floor fractured rough unit is exposed within the same elevation range as the crater floor fractured 1 unit. These observations raise the possibility that the crater floor fractured rough unit could be part of the crater floor fractured 1 unit, as shown in Scenarios 2 and 4, with the two units representing different topographic or erosional expressions of the same bedrock interval. This study’s map also shows a correspondence between occurrences of undifferentiated smooth unit and areas of the crater floor fractured rough unit that appear most topographically distinct from the adjacent crater floor fractured units. This suggests that the previously observed difference in tone between the crater floor fractured rough unit and the crater floor fractured 1 and 2 units was likely the result of the undifferentiated smooth unit overlying large expanses of the crater floor fractured rough unit as a mantle, rather than real tonal difference inherent to the bedrock. The occurrence of undifferentiated smooth unit on the most resistant and topographically distinct expressions of the crater floor fractured rough unit could also suggest a causal relationship between the distribution of the undifferentiated smooth unit and the observed erosional expression of the crater floor units. Perhaps the undifferentiated smooth unit, where it occurred as a mantle, protected and preserved the underlying crater floor fractured units, preferentially shielding some exposures and scarps from erosion. Over time, this mantling effect could have helped to create and enhance the topographic distinctions observed in the crater today.

Scenarios 1 and 2 interpret the fractured units within the crater (crater floor fractured 1 and 2 and margin fractured) to be part of a conformable depositional sequence that is potentially coeval and correlative with the Nili Planum fractured unit outside the crater, consistent with the earlier interpretations of Ehlmann et al. (2008) and Goudge et al. (2015). Here, such a correlation is supported primarily by textural and tonal similarities between the fractured units observed on the crater rim, margin, and outside the crater, and the lack of distinct or distinguishable contacts where contextual and geographic transitions occur. However, Horgan et al. (2020) raised the possibility that fractured units located around the inner margin of the crater (this study’s margin fractured unit) could be lacustrine in origin and time equivalent to delta deposition within the ancient Jezero lake. Scenario 3 acknowledges this possibility by showing the delta units interfingered with the margin fractured unit (Fig. 16). Such an interfingering relationship between the delta and margin fractured unit is geologically plausible in a setting in which the margin fractured unit records a shallow lacustrine facies deposited at the same time the delta formed within the Jezero crater lake basin.

Scenario 4 goes further, suggesting a lacustrine interpretation for all fractured units within Jezero, and a sequence-scale interfingering relationship between the delta and fractured units (Fig. 17). Scenario 4’s interfingering relationship between the delta and the Jezero fractured units (Fig. 17) includes chronostratigraphic elements (i.e., flooding surfaces and time-equivalent facies) known to be present in lake-delta sequences on Earth. Such a scenario may represent the development and evolution of a lake-delta sequence more realistically than the layer-cake unit sequences shown in Scenarios 1 and 2.

Along the inner rim of Jezero, the margin fractured unit extends $\sim200~\mbox{m}$ higher in elevation than the current upper surface of the western delta. If the delta and the margin fractured unit are interfingered as in Scenario 3 (Fig. 16), there was likely a several hundred meter-thick sequence of delta deposits above the present-day surface of the Jezero delta representing the time-equivalent deltaic facies for these stratigraphically younger, higher elevation margin fractured exposures. Eroding this several hundred meter-thick sequence of delta deposits over hundreds of millions to billions of years is perhaps not problematic. However, a mechanism or process capable of producing the inverted topography of the delta at exactly the level at which it is observed today, while the delta deposits that once overlain the present-day delta were easily eroded away, is less obvious. Still, scenarios featuring interfingering relationships between the Jezero infill deposits are geologically plausible and worth considering, particularly given the astrobiological implications of a preserved marginal lacustrine deposit in Jezero crater (Horgan et al. 2020).

### Implications for the Mars 2020 Perseverance Rover Mission

Further examination of the orbiter images and topographic data, as well as orbiter spectroscopic mineralogy data not included in this mapping effort, may help future studies to distinguish between, and ultimately choose, a favored stratigraphic scenario amongst the four presented here. At the present time, and based on this study’s map, we maintain the feasibility of all four scenarios. Each of these scenarios has important implications for the relative timing, duration, origin, habitability, and biosignature preservation potential of the geologic units present in and around the Perseverance field site. The geologic and stratigraphic framework laid out in this study will inform *in situ* sampling decisions and exploration strategies for Perseverance, in addition to providing the field context for samples when, and if, they are returned to Earth.

One major uncertainty highlighted by the four scenarios presented here is the age of the Jezero delta relative to the other infilling units within the crater. Scenarios 1 and 2 propose a relatively young age for the Jezero delta compared to crater floor and margin fractured units, while Scenarios 3 and 4 interpret the western Jezero delta as coeval or older than some of the other units infilling Jezero. Although absolute age dating of samples returned to Earth may eventually provide the sequence of depositional events in Jezero, it will be important to use the Perseverance science payload to document the facies characteristics and cross-cutting and relative age relationships of the delta deposits and the units with which they are in contact. If the margin and crater floor fractured units within Jezero are found to be lacustrine in origin, Scenarios 3 and 4 may emerge as the favored scenarios. If the margin fractured unit is a shallow lacustrine deposit, but the crater floor fractured 1 and 2 units have a different origin, such as a volcanic, Scenario 3 may be the most reasonable correlation of units. Scenarios 3 and 4 are particularly compelling from an astrobiological perspective as they imply the presence of diverse, potentially long-lived proximal and distal subaqueous habitable environments within ancient lake Jezero. Conversely, if the fractured units infilling Jezero are volcanic or aeolian in origin and show no indication of having been deposited in a standing body of water, Scenarios 1 and 2, which propose major unconformities between these units and the delta, may be the most likely. Although the presence of thick sequences of volcanic or aeolian deposits within Jezero may be less compelling from an astrobiological perspective, a volcanic ash, in particular, would be a valuable and highly desired sampling target for the purposes of absolute age dating and geochronology upon the samples’ return to Earth.

Transects by Perseverance across the contacts between the delta and crater floor fractured units, between the delta and the margin fractured unit, and between the remnant mounds and the crater floor fractured units are likely to provide important insights into the relative age of the Jezero delta. Context imagers like Mastcam-Z (Bell et al. this issue) and Navcam (Maki et al. this issue) will provide important documentation of the nature of these contacts, e.g., abrupt versus gradational, but RIMFAX (Hamran et al. this issue), with its ability to penetrate 10–20 m into the subsurface, may be most helpful in distinguishing between onlap versus through-going unit contacts.

Another major unresolved question in Jezero’s geologic history is the origin and relationship between the fractured units inside and outside of Jezero: are they all part of the same depositional sequence with a shared origin, or does each fractured unit represent a distinct depositional process, setting, and age? A thorough investigation of each of the fractured units inside Jezero (crater floor fractured 1 and 2, crater floor fractured rough, and margin fractured) and outside Jezero (Nili Planum fractured unit) with the rover’s arm and mast instruments will reveal similarities or differences in texture, geochemistry, and mineralogy that can be used to address this question. A continuous traverse within Jezero across the transition between the crater floor fractured units and the margin fractured unit will allow the use of RIMFAX and context imagers to document the nature of these contacts.

This study’s geologic map also provides new and updated detail regarding the geologic diversity of the Perseverance field site at Jezero crater and the likely locations at which diverse geologic outcrops will be exposed and accessed to the rover. The improved understanding of the distribution of surficial units throughout the landing ellipse resulting from this mapping effort will help to inform the selection of the best exposed outcrops that are relatively free of cover (dust, sand, lags) that might otherwise have obscured important geologic contacts or relationships. This may be particularly useful in consideration of how to explore the southern portion of the western Jezero delta, which is covered by mantling deposits and extensive fields of aeolian bedforms.

## Conclusions

During the year before launch of the Mars 2020 Perseverance rover mission, the Mars 2020 Science Team undertook an effort to create a photogeologic map of the Perseverance landing ellipse and surrounding area in western Jezero crater using an image mosaic base map and digital terrain model derived from HiRISE data. Sixty-three members of the Mars 2020 Science Team mapped $1.2~\mbox{km}\times 1.2~\mbox{km}$ quadrangles at 1:5000 digital map scale. Main results of the mapping effort are summarized below:

1. Bedrock and surficial units observed throughout the landing site are grouped by crater floor, delta, margin, crater rim, Neretva Vallis and Nili Planum settings. Bedrock units identified in this study were generally consistent with those identified in previously published mapping efforts, but this contribution mapped the delta and distribution of surficial units more completely and at a higher level of detail than previous studies.
2. The floor of Jezero crater was mapped as three distinct bedrock units, although portions of the floor were recognized as covered by an undifferentiated smooth mantle and extensive fields of aeolian bedforms. Despite previous interpretations—particularly Schon et al. (2012) and Goudge et al. (2015)—no evidence for lava flows was found.
3. Four units were mapped on the western Jezero delta and in mounds interpreted to be remnants of a more formerly extensive deltaic or lacustrine deposit, including (from oldest to youngest), the delta thinly layered unit, thickly layered unit, truncated curvilinear layered unit, and blocky units observed on the delta top. A fifth unit, the delta layered rough unit, was mapped in an outcrop to the northeast of the western Jezero delta, although no evidence was observed to either support or refute a connection between this deposit and Sava Vallis, as has been suggested by previous studies.
4. The deposit occurring along the inner margin of Jezero crater was mapped as a single unit, the margin fractured unit. Although a variety of textures—high vs. low relief, blocky vs. smooth, fractures—were recognized within it, these variable surface expressions could not be consistently mapped as units representing rock volumes, so further subdivision was not attempted. Fractured units within Jezero were mapped separately from those present outside the crater, although they appear morphologically similar due to their light tone, lack of clear layering, and abundant polygonal fractures.
5. The Jezero crater rim is composed predominantly of a rough, rubbly blocky unit with intermittent exposures of layered, fractured, and brecciated outcrop.
6. A layered unit was observed in the walls and floor of Neretva Vallis, distinct from deposits found within Jezero or on Nili Planum. This unit is interpreted to be related to fluvial and/or lacustrine activity within the channel and outside the crater.
7. Four possible relative age correlations for the mapped bedrock units are presented to explain the relative age relationships of major units within the map area. One is generally consistent with previous published interpretations, but the others consider more complex interfingering relationships between the western Jezero delta and adjacent units, or alternative interpretations of the relative age relationships of the main mapped units. Further analysis of orbiter data, investigation on the ground by the Mars 2020 Perseverance rover, and possibly laboratory analysis of returned samples, are likely needed to distinguish between these different scenarios.

## Supplementary Information

Below are the links to the electronic supplementary material.

High Resolution Imaging Science Experiment (HiRISE) image pairs used to construct the HiRISE base map and HiRISE digital terrain model used in this study and links to repositories hosting these basemaps. (PDF 171 kB)

Mapping quadrangles with informal quad names and the Perseverance landing ellipse displayed on the HiRISE basemap. (TIF 18.6 MB)

GIS-ready shapefile, associated auxiliary files, and README file containing the Mars 2020 Science Team’s photogeologic map of the Perseverance rover landing site in Jezero crater. (ZIP 3.3 MB)

## Data Availability

The HiRISE image pairs (listed in Online Resource 1, ESM_1.txt) that comprise the HiRISE visible base map used in this study are available online at the Astropedia lunar and planetary catographic catalog: https://planetarymaps.usgs.gov/mosaic/mars2020_trn/HiRISE/. The HiRISE visible base map is available at: https://astrogeology.usgs.gov/search/map/Mars/Mars2020/JEZ_hirise_soc_006_orthoMosaic_25cm_Eqc_latTs0_lon0_first. The HiRISE digital terrain model that was used to produce the slope map, stereo anaglyph, artificial hillshade, colorized shaded relief, and topographic contours at 1, 5, 10, 20, 50, and 100 meter intervals used in this study can be accessed at: https://astrogeology.usgs.gov/search/ map/Mars/Mars2020/JEZ_hirise_soc_006_DTM_MOLAtopography_DeltaGeoid_1m_Eqc_latTs0_lon0_ blend40. Mapping shapefiles are included as Online Resource 3 (ESM_3.zip). The CRISM MTRDR false color basemap can be accessed here: https://data.nasa.gov/docs/datasets/public/CRISM-Mosaic/jezero_crater_mosaic_SET_OPT_TAN_rect_flightCTX.tfw.

## Code availability

Source files for the CAMP tool, “Web-based Spatial Data Infrastructure for Planetary Science Operations” are available on GitHub at https://github.com/NASA-AMMOS/MMGIS.
