# Supplementary material for: Photogeologic Map of the Perseverance Rover Field Site in Jezero Crater Constructed by the Mars 2020 Science Team
Source: Space Sci Rev. 2020 Nov 3;216(8):127. doi: 10.1007/s11214-020-00739-x (PMC7116714; doi:10.1007/s11214-020-00739-x)
Supplement: Supplementary file 1 — High Resolution Imaging Science Experiment (HiRISE) image pairs used to construct the HiRISE base map and HiRISE digital terrain model used in this study and links to repositories hosting these basemaps. (PDF 171 kB) [file 11214_2020_739_MOESM1_ESM.pdf]

Article Title: Photogeologic Map of the Perseverance Rover Field Site in Jezero Crater Constructed by the Mars 2020 Science Team

Journal: Space Science Reviews

Kathryn M. Stack (0000-0003-3444-6695)[1], Nathan R. Williams[1], Fred Calef III (0000-0002-5132-3980)[1], Vivian Sun (0000-0003-1480-7369)[1], Kenneth H. Williford (0000-0003-0633-408X)[1], Kenneth A. Farley (0000-0002-7846-7546)[2], Sigurd Eide[3], David Flannery (0000-0001-8982-496X)[4], Cory Hughes (0000-0002-7061-1443)[5], Samantha R. Jacob (0000-0001-9950-1486)[6], Linda C. Kah (0000-0001-7172-2033)[7], Forrest Meyen (0000-0002-0140-6411)[8], Antonio Molina (0000-0002-5038-2022)[9], Cathy Quantin Nataf[10], Melissa Rice (0000-0002-8370-4139)[4], Patrick Russell[11], Eva Scheller (0000-0002-9981-5802)[2], Christina H. Seeger[5], William J. Abbey[1], Jacob B. Adler (0000-0002-4722-2909)[12], Hans Amundsen[13], Ryan B. Anderson (0000-0003-4465-2871)[14], Stanley M. Angel (0000-0002-0328-0568)[15], Gorka Arana (0000-0001-7854-855X)[16], James Atkins[7], Megan Barrington[17], Tor Berger[18], Rose Borden (0000-0002-2908-598X)[7], Beau Boring[7], Adrian Brown[19], Brandi L. Carrier (0000-0001-9943-7138)[1], Pamela Conrad (0000-0001-5724-3343)[20], Henning Dypvik[2], Sarah A. Fagents (0000-0002-3943-3335)[21], Zachary E. Gallegos[22], Brad Garczynski[23], Keenan Golder (0000-0002-4968-2239)[7], Felipe Gomez (0000-0001-9977-7060)[9], Yulia Goreva[1], Sanjeev Gupta[24], Svein-Erik Hamran[3], Taryn Hicks[7], Eric D. Hinterman[25], Briony N. Horgan (0000-0001-6314-9724)[23], Joel Hurowitz[26], Jeffrey R. Johnson[27], Jeremie Lasue (0000-0001-9082-4457)[28], Rachel E. Kronyak[1], Yang Liu[1], Juan Manuel Madariaga (0000-0002-1685-6335)[16], Nicolas Mangold[29], John McClean[24], Noah Miklusicak[7], Daniel Nunes (0000-0002-0937-7176)[1], Corrine Rojas[6], Kirby Runyon (0000-0001-6361-6548)[27], Nicole Schmitz[31], Noel Scudder[23], Emily Shaver[7], Jason SooHoo (0000-0003-1938-0720)[25], Russell Spaulding[7], Evan Stanish[31], Leslie K. Tamppari[1], Michael M. Tice[32], Nathalie Turenne[31], Peter A. Willis (0000-0001-5394-1101)[1], R. Aileen Yingst (0000-0002-0628-4265)[33]

[1]*Jet Propulsion Laboratory, California Institute of Technology, 4800 Oak Grove Drive, Pasadena, CA 91109*
[2]*California Institute of Technology, Pasadena, CA*
[3]*University of Oslo, Oslo, Norway*
[4]*Queensland University of Technology, Brisbane, Queensland, Australia*
[5]*Western Washington University, Bellingham,WA*
[6]*Arizona State University, Tempe, Arizona*
[7]*University of Tennessee-Knoxville, Knoxville, TN*
[8]*Draper Laboratory, Cambridge, MA*
[9]*Centro de Astrobiología, CAB (INTA, CSIC), Madrid, Spain*
[10]*University of Lyon, Lyon, France*
[11]*University of California Los Angeles, Los Angeles, CA*
[12]*Johns Hopkins University, Baltimore, MD*
[13]*Earth and Planetary Exploration Services, Berlin, Germany*
[14]*USGS-Flagstaff, Flagstaff, AZ*
[15]*University of South Carolina, Columbia, SC*
[16]*University of the Basque Country (UPV/EHU), Leioa, Bizkaia, Spain*
[17]*Plancius Research, Severna Park, MD*
[18]*Carnegie Institution for Science, Washington, D.C.*
[19]*Cornell University, Ithaca, NY*
[20]*Forsvarets forskingsinstitutt, Kjeller, Norway*
[21]*University of Hawaii at Manoa, Honolulu, HI*

[22]*University of New Mexico, Albuquerque, NM*
[23]*Purdue University, West Lafayette, IN*
[24]*Imperial College of London*
[25]*Massachusetts Institute of Technology, Cambridge, MA*
[26]*State University of New York- Stony Brook, Stony Brook, NY*
[27]*Johns Hopkins Applied Physics Laboratory, Laurel, MD*
[28]*Institut de Recherche en Astrophysique et Planetologie (IRAP), Université de Toulouse, Paul Sabatier, Toulouse, France*
[29]*Laboratoire Planétologie et Géodynamique, UMR 6112, CNRS, Université de Nantes, Nantes, France*
[30]*Deutsches Zentrum Fuer Luft- und Raumfahrt E.V., Cologne, Germany*
[31]*University of Winnipeg, Winnipeg, Manitoba, CA*
[32]*Texas A&M University, College Station, TX*
[33]*Planetary Science Institute, Tucson, AZ*

Corresponding author:
Kathryn M. Stack
kathryn.m.stack@jpl.nasa.gov
ORC-ID 0000-0003-3444-6695
Jet Propulsion Laboratory
M/S 321-400
4800 Oak Grove Drive
Pasadena, CA 91109

Copyright 2020. All rights reserved.

High Resolution Imaging Science Experiment (HiRISE) image pairs used to construct the HiRISE basemap and HiRISE digital terrain model:

ESP_045994_1985	ESP_046060_1985
PSP_002387_1985	PSP_003798_1985
ESP_037396_1985	ESP_042315_1985
ESP_048842_1985	ESP_048908_1985
ESP_037818_1990	ESP_037330_1990
ESP_036618_1985	ESP_037119_1985

The HiRISE basemap used in this study can be accessed here:
https://planetarymaps.usgs.gov/mosaic/mars2020_trn/HiRISE/JEZ_hirise_soc_006_orthoMosaic_25cm_Eqc_latTs0_lon0_first.tif

The HiRISE digital terrain model mosaic used in this study can be accessed here:
https://planetarymaps.usgs.gov/mosaic/mars2020_trn/HiRISE/JEZ_hirise_soc_006_DTM_MOLAtopography_DeltaGeoid_1m_Eqc_latTs0_lon0_blend40.tif
