# Supplementary figures and images for: Photogeologic Map of the Perseverance Rover Field Site in Jezero Crater Constructed by the Mars 2020 Science Team
Source: Space Sci Rev. 2020 Nov 3;216(8):127. doi: 10.1007/s11214-020-00739-x (PMC7116714; doi:10.1007/s11214-020-00739-x)

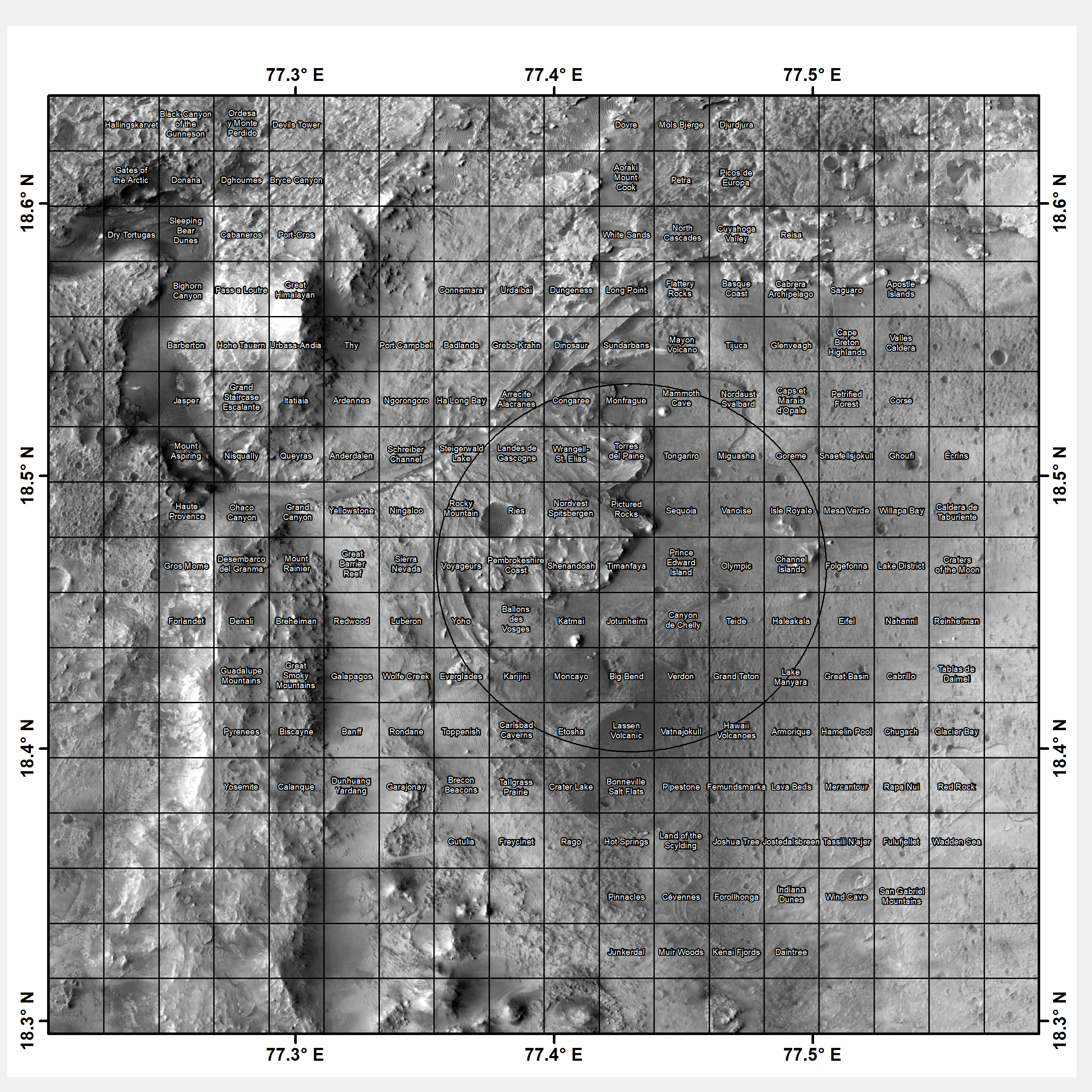

Supplement: Supplementary file 2 — Mapping quadrangles with informal quad names and the Perseverance landing ellipse displayed on the HiRISE basemap. (TIF 18.6 MB) [file 11214_2020_739_MOESM2_ESM.tif]
