# Supplementary material for: Photogeologic Map of the Perseverance Rover Field Site in Jezero Crater Constructed by the Mars 2020 Science Team
Source: Space Sci Rev. 2020 Nov 3;216(8):127. doi: 10.1007/s11214-020-00739-x (PMC7116714; doi:10.1007/s11214-020-00739-x)
Supplement: Supplementary file 3 — GIS-ready shapefile, associated auxiliary files, and README file containing the Mars 2020 Science Team’s photogeologic map of the Perseverance rover landing site in Jezero crater. (ZIP 3.3 MB) [file 11214_2020_739_MOESM3_ESM.zip › EMS_3_README.pdf]

Article Title: Photogeologic Map of the Perseverance Rover Field Site in Jezero Crater Constructed by the Mars 2020 Science Team

Journal: Space Science Reviews

Kathryn M. Stack (0000-0003-3444-6695)[1], Nathan R. Williams[1], Fred Calef III (0000-0002-5132-3980)[1], Vivian Z. Sun (0000-0003-1480-7369)[1], Kenneth H. Williford (0000-0003-0633-408X)[1], Kenneth A. Farley (0000-0002-7846-7546)[2], Sigurd Eide[3], David Flannery (0000-0001-8982-496X)[4], Cory Hughes (0000-0002-7061-1443)[5], Samantha R. Jacob (0000-0001-9950-1486)[6], Linda C. Kah (0000-0001-7172-2033)[7], Forrest Meyen (0000-0002-0140-6411)[8], Antonio Molina (0000-0002-5038-2022)[9], Cathy Quantin Nataf[10], Melissa Rice (0000-0002-8370-4139)[4], Patrick Russell[11], Eva Scheller (0000-0002-9981-5802)[2], Christina H. Seeger[5], William J. Abbey[1], Jacob B. Adler (0000-0002-4722-2909)[12], Hans Amundsen[13], Ryan B. Anderson (0000-0003-4465-2871)[14], Stanley M. Angel (0000-0002-0328-0568)[15], Gorka Arana (0000-0001-7854-855X)[16], James Atkins[7], Megan Barrington[17], Tor Berger[18], Rose Borden (0000-0002-2908-598X)[7], Beau Boring[7], Adrian Brown[19], Brandi L. Carrier (0000-0001-9943-7138)[1], Pamela Conrad (0000-0001-5724-3343)[20], Henning Dypvik[3], Sarah A. Fagents (0000-0002-3943-3335)[21], Zachary E. Gallegos[22], Brad Garczynski[23], Keenan Golder (0000-0002-4968-2239)[7], Felipe Gomez (0000-0001-9977-7060)[9], Yulia Goreva[1], Sanjeev Gupta[24], Svein-Erik Hamran[3], Taryn Hicks[7], Eric D. Hinterman[25], Briony N. Horgan (0000-0001-6314-9724)[23], Joel Hurowitz[26], Jeffrey R. Johnson[27], Jeremie Lasue (0000-0001-9082-4457)[28], Rachel E. Kronyak[1], Yang Liu[1], Juan Manuel Madariaga (0000-0002-1685-6335)[16], Nicolas Mangold[29], John McClean[24], Noah Miklusicak[7], Daniel Nunes (0000-0002-0937-7176)[1], Corrine Rojas[6], Kirby Runyon (0000-0001-6361-6548)[27], Nicole Schmitz[31], Noel Scudder[23], Emily Shaver[7], Jason SooHoo (0000-0003-1938-0720)[25], Russell Spaulding[7], Evan Stanish[31], Leslie K. Tamppari[1], Michael M. Tice[32], Nathalie Turenne[31], Peter A. Willis (0000-0001-5394-1101)[1], R. Aileen Yingst (0000-0002-0628-4265)[33]

[1]*Jet Propulsion Laboratory, California Institute of Technology, 4800 Oak Grove Drive, Pasadena, CA 91109*
[2]*California Institute of Technology, Pasadena, CA*
[3]*University of Oslo, Oslo, Norway*
[4]*Queensland University of Technology, Brisbane, Queensland, Australia*
[5]*Western Washington University, Bellingham,WA*
[6]*Arizona State University, Tempe, Arizona*
[7]*University of Tennessee-Knoxville, Knoxville, TN*
[8]*Draper Laboratory, Cambridge, MA*
[9]*Centro de Astrobiología, CAB (INTA, CSIC), Madrid, Spain*
[10]*University of Lyon, Lyon, France*
[11]*University of California Los Angeles, Los Angeles, CA*
[12]*Johns Hopkins University, Baltimore, MD*
[13]*Earth and Planetary Exploration Services, Berlin, Germany*
[14]*USGS-Flagstaff, Flagstaff, AZ*
[15]*University of South Carolina, Columbia, SC*
[16]*University of the Basque Country (UPV/EHU), Leioa, Bizkaia, Spain*
[17]*Cornell University, Ithaca, NY*
[18]*Forsvarets forskingsinstitutt, Kjeller, Norway*
[19]*Plancius Research, Severna Park, MD*
[20]*Carnegie Institution for Science, Washington, D.C.*
[21]*University of Hawaii at Manoa, Honolulu, HI*

[22]*University of New Mexico, Albuquerque, NM*
[23]*Purdue University, West Lafayette, IN*
[24]*Imperial College of London, London, UK*
[25]*Massachusetts Institute of Technology, Cambridge, MA*
[26]*State University of New York- Stony Brook, Stony Brook, NY*
[27]*Johns Hopkins Applied Physics Laboratory, Laurel, MD*
[28]*Institut de Recherche en Astrophysique et Planetologie (IRAP), Université de Toulouse, Paul Sabatier, Toulouse, France*
[29]*Laboratoire Planétologie et Géodynamique, UMR 6112, CNRS, Université de Nantes, Nantes, France*
[30]*Deutsches Zentrum Fuer Luft- und Raumfahrt E.V., Cologne, Germany*
[31]*University of Winnipeg, Winnipeg, Manitoba, CA*
[32]*Texas A&M University, College Station, TX*
[33]*Planetary Science Institute, Tucson, AZ*

Corresponding author:
Kathryn M. Stack
kathryn.m.stack@jpl.nasa.gov
ORC-ID 0000-0003-3444-6695
Jet Propulsion Laboratory
M/S 321-400
4800 Oak Grove Drive
Pasadena, CA 91109

Copyright 2020. All rights reserved.

GIS-ready shapefile and associated auxiliary files containing the Mars 2020 photogeologic map of the Perseverance landing site in Jezero crater. Files were created with Esri's ArcGIS Pro 2.3 GIS application.
